# Systematic identification and characterization of genes in the regulation and biogenesis of photosynthetic machinery

**DOI:** 10.1016/j.cell.2023.11.007

**Published:** 2023-12-07

**Authors:** Moshe Kafri, Weronika Patena, Lance Martin, Lianyong Wang, Gillian Gomer, Sabrina L. Ergun, Arthur K. Sirkejyan, Audrey Goh, Alexandra T. Wilson, Sophia E. Gavrilenko, Michal Breker, Asael Roichman, Claire D. McWhite, Joshua D. Rabinowitz, Frederick R. Cross, Martin Wühr, Martin C. Jonikas

**Affiliations:** 1 Department of Molecular Biology, Princeton University, Princeton, NJ 08544, USA; 2 Laboratory of Cell Cycle Genetics, The Rockefeller University, New York, NY 10021, USA; 3 Lewis-Sigler Institute for Integrative Genomics and Department of Chemistry, Princeton University, Princeton, NJ 08544, USA; 4 Howard Hughes Medical Institute, Princeton University, Princeton, NJ 08544, USA; 5 These authors contributed equally; 6 Lead contact

## Abstract

Photosynthesis is central to food production and the Earth’s biogeochemistry, yet the molecular basis for its regulation remains poorly understood. Here, using high-throughput genetics in the model eukaryotic alga *Chlamydomonas reinhardtii*, we identify with high confidence (false discovery rate [FDR] < 0.11) 70 poorly characterized genes required for photosynthesis. We then enable the functional characterization of these genes by providing a resource of proteomes of mutant strains, each lacking one of these genes. The data allow assignment of 34 genes to the biogenesis or regulation of one or more specific photosynthetic complexes. Further analysis uncovers biogenesis/regulatory roles for at least seven proteins, including five photosystem I mRNA maturation factors, the chloroplast translation factor MTF1, and the master regulator PMR1, which regulates chloroplast genes via nuclear-expressed factors. Our work provides a rich resource identifying regulatory and functional genes and placing them into pathways, thereby opening the door to a system-level understanding of photosynthesis.

## In brief

Kafri et al. used a genetic screen to identify 70 previously uncharacterized genes required for photosynthesis in the model alga *Chlamydomonas reinhardtii*. Using mutant proteome profiling, they assign many of the genes to pathways, revealing biogenesis and regulatory factors including the master regulator PMR1, which regulates chloroplast genes via nuclear-expressed factors.

## Graphical Abstract

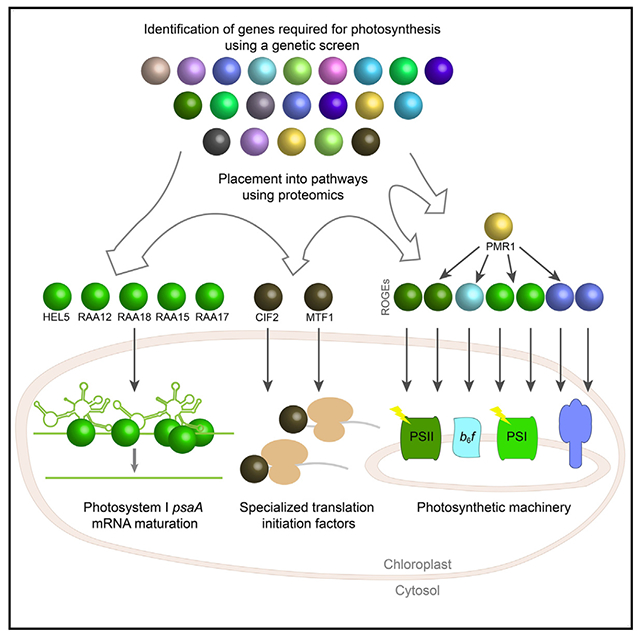

## INTRODUCTION

**Figure 1.**
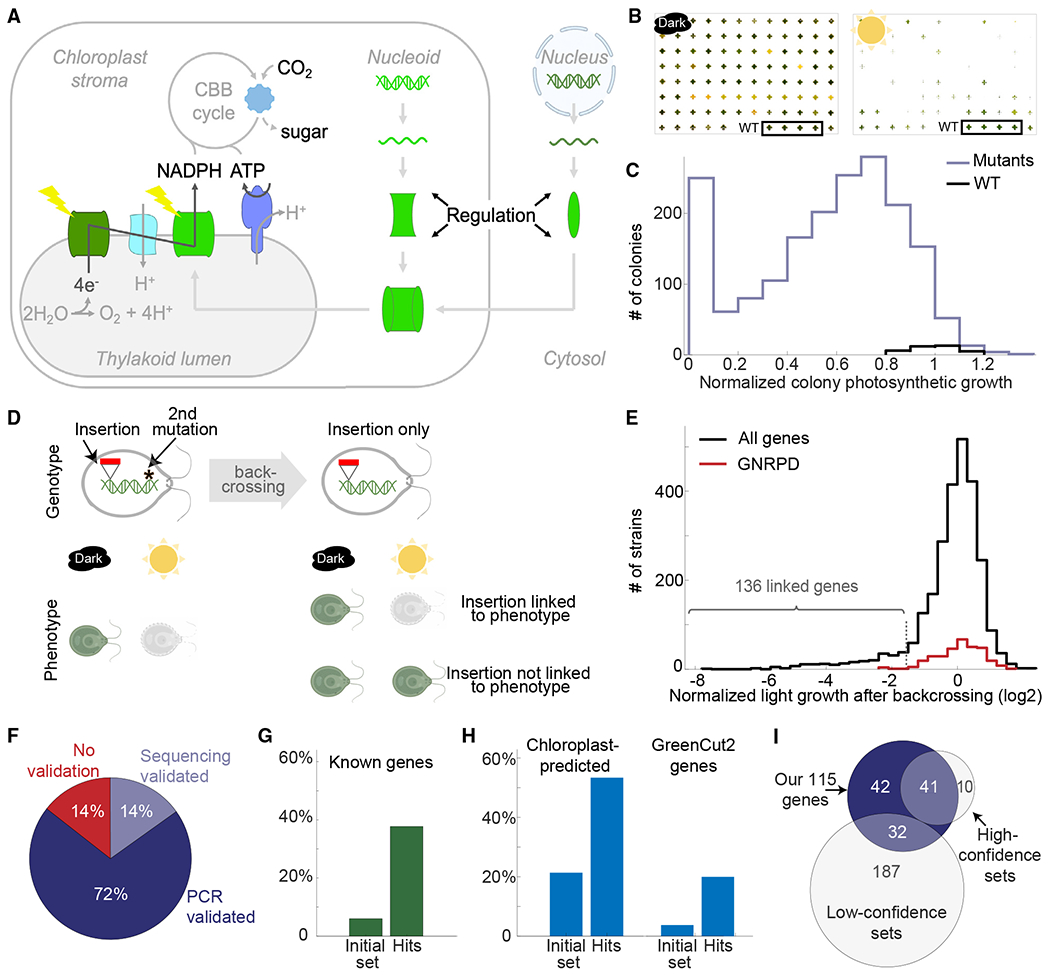
Identification of 115 genes required for photosynthesis (A) Schematic of biogenesis and regulation of the photosynthesis machinery. (B) Photosynthetic growth phenotype validation for 1,781 previously identified photosynthesis-deficient *Chlamydomonas* mutants.^25^ Photosynthesis-deficient mutants can grow in the dark with acetate but have growth defects in 750 μE/m^2^/s light without acetate (wild type [WT]). See also Figure S3A. (C) Normalized colony photosynthetic growth for different mutants (blue) or WT (black). Growth was measured using a metric that incorporates colony size and color (see STAR Methods). Shown is the median of 4 replicates. (D) Most of the mutant strains have second-site mutations that could cause the photosynthetic phenotype. We used backcrossing to allow segregation between the insertion and second-site mutations. For higher throughput, we developed a pooled backcrossing method (Figure S1). (E) Histogram of normalized light growth after backcrossing for all strains (black) and for strains disrupted in “genes whose disruption likely did not result in a photosynthesis defect” (GNRPD, STAR Methods, red). Mutants disrupted in 136 genes showed normalized light growth after backcrossing below the threshold of 0.34 (−1.55 on a log_2_ scale). These genes are linked to the phenotype with FDR < 0.11 (Figure S1). (F) Validation of the insertion mapping of ~86% of the candidates using PCR and sequencing (see also Figure S2). (G) Approximately 39% of our hits had a previously known role in photosynthesis (29 in *Chlamydomonas* and 16 in land plant homologs), compared with 6% in the initial set. (H) The hits are enriched in chloroplast-predicted proteins (PredAlgo^30^) and in GreenCut2 green lineage-specific genes.^10^ (I) Our 115 photosynthetic hits captured most of the previously identified high-confidence hits (41 of 51) and increased the confidence of ~14% of the previously low-confidence hits (32 of 219) (STAR Methods; Figure S1G).

In photosynthetic eukaryotes, the photosynthetic apparatus consists of a series of protein complexes in the chloroplast thylakoid membrane that use light energy to produce NADPH, ATP, and other cellular energy carriers.^1^ NADPH and ATP, in turn, power many pathways, notably CO_2_ assimilation into sugar by the Calvin-Benson-Bassham metabolic cycle^2^ (Figure 1A).^2^

As a sophisticated system central to cellular fitness, hundreds of genes encoded in both the nucleus and chloroplast are required to assemble these complexes^3^ and regulate their activity^3^ under nuclear control.^4^ In plants and green algae, this coordination is known to involve a range of different mechanisms, including post-transcriptional regulation of chloroplast-expressed genes by nuclear-encoded proteins,^5^ translational regulation of chloroplast-expressed subunits by assembly intermediates of photosynthetic complexes,^6^ and protease-mediated degradation of unassembled subunits.^7^

Although photosynthesis and its regulation have been extensively studied for 70 years,^8,9^ phylogenetics suggests that hundreds of genes participating in photosynthesis remain to be identified and characterized. Indeed, approximately half of the GreenCut2 genes—a set of 597 genes conserved only in the green photosynthetic eukaryotic lineage and therefore likely to be involved in photosynthesis^10^—have not been functionally characterized.

Genetic screens have been done in land plants and algae to identify missing photosynthesis genes. Land plant screens have identified photosynthesis-deficient mutants based on leaf coloration,^11,12^ seedling lethality,^13^ and chlorophyll fluorescence.^14,15^ As a complementary system to plants, the leading unicellular model eukaryotic alga *Chlamydomonas reinhardtii* (*Chlamydomonas*) has provided advantages of higher throughput and physiology that facilitate the identification and characterization of genes essential to photosynthesis.^16,17^ These characteristics have been leveraged to identify and characterize many core components of the photosynthetic electron transport chain.^18–20^

In the past decade, several hundred candidates for genes involved in photosynthesis have been uncovered by screens of two large *Chlamydomonas* mutant collections, Niyogi CAL^21–23^ and CLiP.^24,25^ However, these screens had many false positives and there are indications that fewer than half of these candidates are actually involved in photosynthesis.^25^ Current challenges facing the field include (1) determining which of these candidates are genuinely involved in photosynthesis and (2) determining the functions of validated photosynthesis genes.

Here, we address these two challenges by combining genetics and proteomics to identify and functionally characterize genes required for photosynthesis with high confidence on a global scale. We first identified with high confidence (false discovery rate [FDR] < 0.11) a total of 115 genes required for photosynthesis–including 70 genes whose molecular function in photosynthesis had not been previously characterized in any organism–by confirming linkage of each mutation with the observed photosynthetic defect and validating insertion site mappings. We then determined the proteomic profiles of mutants representing these genes to initiate their functional characterization, including assigning 34 of them to specific photosynthetic pathways. As proof of principle for the utility of our resource, we performed additional analyses, which revealed that five of these factors work with known factors to regulate mRNA maturation of key photosystem I (PSI) subunit PsaA. We also discovered and characterized two post-transcriptional regulators of photosynthetic apparatus biogenesis, providing insights into how cells leverage the chloroplast translation machinery and the regulation of nuclear gene expression to control photosynthetic complex abundance. Together, our dataset opens the door to rapid characterization of photosynthesis genes and provides systems-level insights into photosynthesis regulation.

## RESULTS

### A framework for high-confidence identification of genes with roles in photosynthesis

Previous large-scale *Chlamydomonas* screens suffered from the limitation that most mutant strains carried mutations in multiple genes,^21,25^ preventing high-confidence identification of the specific gene whose disruption causes the observed photosynthetic defect unless multiple independent mutants in the same gene showed the same defect.^24,25^ Here, we overcame this limitation by developing a high-throughput implementation of traditional genetic linkage analysis between a mutation and an observed photosynthetic defect, which allowed us to identify with high confidence the specific gene whose disruption is responsible for the defect, even if that gene was disrupted in only one mutant.

### Pooled backcrossing and mapping validation of putative photosynthetic genes

We started this study with a set of 1,781 mapped random-insertion mutants from the CLiP library of *Chlamydomonas* mutants that we previously identified to have a photosynthetic growth defect.^25^ We first validated the mutants’ phenotypes using an automated spot test on agar (Figures 1B and 1C; STAR Methods).

To determine whether a given mapped insertion or another unknown mutation was the cause of the observed photosynthetic defect, we determined whether the insertion was genetically linked to the defect using backcrossing. Backcrossing involves mating a mutant of interest with a wild-type (WT) strain and analyzing the progeny. This process results in random segregation of the different mutations present in the original mutant strain, thereby allowing the impact of each mutation on the phenotype of interest— in our case, defective photosynthetic growth—to be separated. If all progeny carrying a particular insertion exhibited a defect in photosynthetic growth, we concluded that the insertion is genetically linked to the defect, indicating that the disruption of the gene likely caused the defect (Figure 1D).

To overcome the limited throughput (~10 mutants per experiment) of traditional backcrossing, we developed a pooled method that allowed us to backcross nearly 1,000 mutants per experiment (Figure S1A; STAR Methods; Breker et al.^26^). We backcrossed pools of hundreds of mutants and then grew the pooled progeny under photosynthetic and heterotrophic conditions. We determined the relative abundance of each insertion after growth under each condition by sequencing the unique DNA barcode(s) associated with that insertion^25^ (Figure 1E; Table S1; STAR Methods). Depletion of a barcode in the photosynthetic condition pool indicated linkage of the corresponding insertion to the photosynthesis defect.

We sought to estimate the frequency of incorrect identification of causal genes in this approach. Such errors could arise in rare cases where the insertion is not causal but merely in the genomic vicinity of the causal mutation or could be due to measurement noise. We quantified the frequency of such errors with a FDR metric. To calculate the FDR, we used a set of genes whose disruption likely did not result in a photosynthesis defect and measured their prevalence among our hits (Figures 1E and S1B–S1F; STAR Methods). This calculation identified 227 genes linked to a photosynthetic defect with an FDR of 0.3. Using a stricter threshold (light/dark abundance ≦ 0.34; Figure 1E), we identified 136 genes with FDR < 0.11 (Figures 1E, S1C, and S1D; Table S1); we continued with this set for further analysis. 27 of these 136 genes were represented by two or more independent linked insertions, providing further support of their roles in photosynthesis.

Some of the insertions from the starting collection of 1,781 mutants are known to be mapped to incorrect sites in the genome.^25^ Therefore, we validated the mapping of our linked insertions using colony PCR (Figure S2) or whole-genome sequencing (Figures 1F and S2; Table S1; STAR Methods). Altogether, we identified with high fidelity 115 genes required for photosynthesis from our initial set of 1,781 photosynthesis-deficient mutants (Figure S2A).

Approximately 40% of the 115 genes have a known role in photosynthesis in *Chlamydomonas* (29 genes) or in land plants (16 genes) (Figure 1G), a substantial enrichment compared with ~6% of the genes in the initial 1,781 mutants. The 115 genes are also enriched in metrics associated with photosynthesis: they show a 2.5-fold enrichment in predicted localization to the chloroplast^27^ and a 4-fold enrichment in genes conserved specifically in the green lineage^10^ (Figure 1H).

A subset of our data provides orthogonal validation of candidate photosynthesis genes. Our 115 genes required for photosynthesis include 41 of the 51 genes identified with high confidence (FDR < 0.3) in previous large-scale photosynthesis screens based on the CLiP mutant collection^24,25^ (Figure 1I). This high overlap shows the quality of both datasets. Our 115 genes also include 32 of 219 genes that were previously low-confidence candidates (no FDR was calculated) in the CLiP and Niyogi CAL collections (Figure 1I), increasing the confidence that these 32 genes do indeed participate in photosynthesis. Of the remaining 42 genes, 38 had not previously been identified as being required for photosynthesis in any organism.

Altogether, our 115 genes included 70 genes whose molecular function in photosynthesis had not been previously characterized in any organism (Figure S2A). We have noted in Table S1 additional information from other sources that further supports or weakens our confidence in their involvement in photosynthesis. The study of these genes represents a new frontier for photosynthesis research.

### Hit validation and protein localization

**Figure 2.**
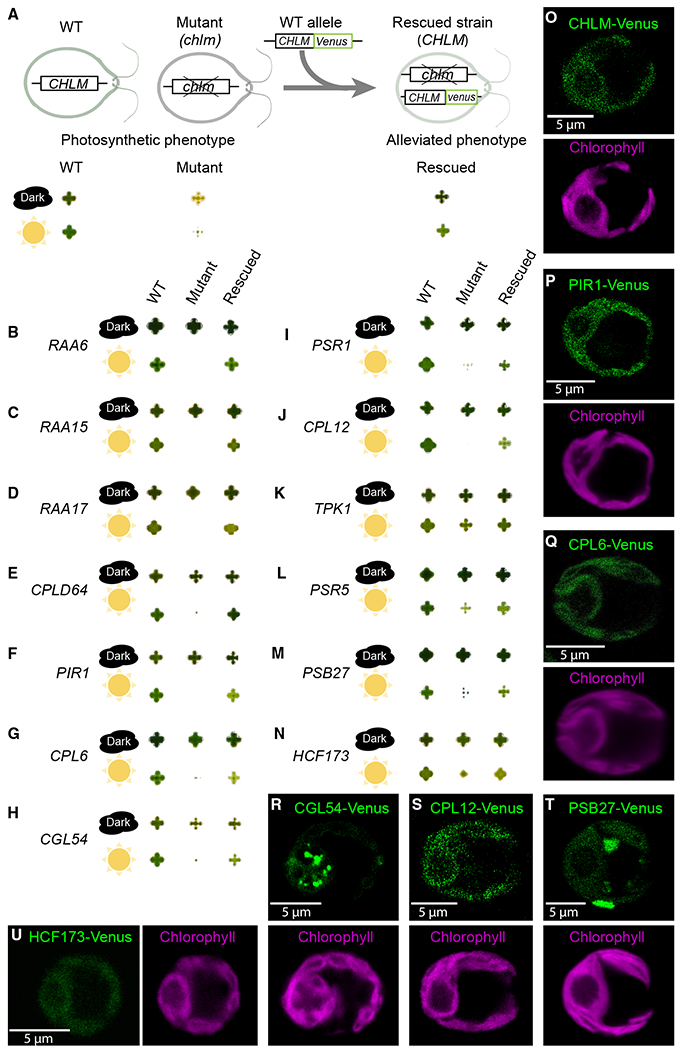
Gene rescue and protein localization (A) Schematic of the genetic rescue procedure for the known chlorophyll biosynthesis gene *CHLM*. In the dark with acetate, *chlm* grows almost as well as wild type but is yellow^44^; under high light, the mutant has a severe growth defect. Transformation of the mutant with a Venus-tagged *CHLM* alleviates both the color and growth phenotypes. (B–N) The colony growth of wild type, mutants, and the mutants we rescued by transforming with the wild-type genes. (See also STAR Methods and Figure S3B.) (O) Localization of CHLM-Venus in the wild-type background. A similar localization was observed in the rescued strain. (P–U) Localizations of Venus-tagged proteins. CPL6, CGL54, and HCF173 are in the mutant background; PIR1, CPL12, and PSB27 are in the wild-type background due to insufficient expression in the rescued mutant strain.

To experimentally validate the involvement of the genes we identified in photosynthesis, we sought to genetically rescue the photosynthetic defect of the mutants that have insertions in genes not previously known to function in photosynthesis. Gene rescue involves testing whether transforming a mutant with a WT copy of the gene alleviates the phenotype (Figure 2A). Despite challenges to gene rescue in *Chlamydomonas* due to difficulties with PCR amplification and expression of heterologous genes,^28–30^ we rescued mutants in 16 genes out of 36 that we attempted. Considering the low efficiency of construct expression in *Chlamydomonas*,^30,31^ this success rate is close to the maximum that would be expected even if all 36 genes were required for photosynthesis.^30,31^ The genes whose functions in photosynthesis were validated by mutant rescue included 12 genes that had not previously been implicated in photosynthesis in any organism (Figures 2, 6C, and 6H; Table 1) and two genes whose function in photosynthesis had not previously been characterized in *Chlamydomonas* (Figure 2; Table S2).

Nine of the 16 rescued mutants showed sufficient expression to allow us to use the C-terminal fluorescent Venus tag in the rescue construct to determine protein localization (Figures 2O–2U, 6D, 6P, S3D, and S3E). While two of these proteins exhibited dual localizations (Figures 2T and 6P), in every case a significant portion of the protein localized to the chloroplast, consistent with their putative role in photosynthesis.

Based on the literature (Table 1) and our data (Table 1 and analyses below), we suggest that of the 12 rescued genes not previously known to be required for photosynthesis, at least four are post-transcriptional regulation factors (RAA17, RAA15, photosynthesis master regulator 1 [PMR1], and methionyl-tRNA formyltransferase 1 [MTF1]), four are biogenesis or repair factors for the photosynthetic apparatus (CPLD64, photosystem I required 1 [PIR1], CPL6, and CGL54), and three play roles in metabolism (PSR1, CPL12, and TPK1). The validation of these genes illustrates how much remains to be learned about photosynthesis and underscores the quality and value of our high-confidence list of genes as a starting point for studying lesser-known areas of photosynthesis.

### Mutant proteomic profiling informs gene function

**Figure 3.**
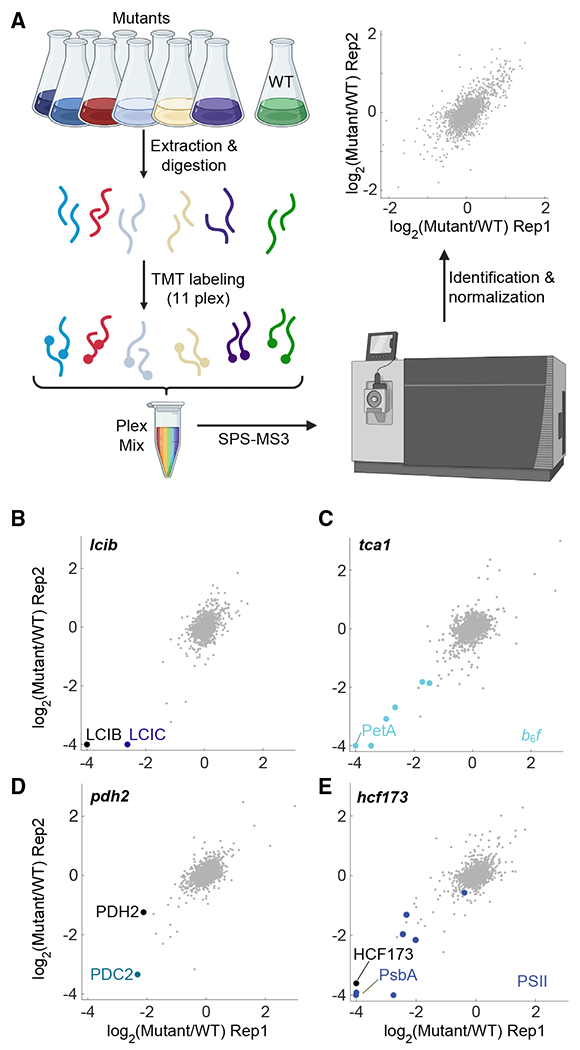
Proteomic data reproduce known phenotypes and validate predicted phenotypes (A) In each experiment, ten mutant strains and a wild-type control were grown under dark conditions. After extraction and digestion, we labeled peptides with tandem mass tags (TMTs) and analyzed them using SPS-MS3 mass spectrometry. At least two independent experiments were carried out for each mutant (STAR Methods). The normalized log_2_ of mutant/WT protein abundance in two replicates is plotted. (B) LCIB and LCIC protein abundances are shown in the *lcib* mutant. (C) Cytochrome *b*_6_*f* protein subunit abundances are shown in the *tca1* mutant. (D) Abundance of predicted *Chlamydomonas* pyruvate dehydrogenase E1 alpha subunit PDC2 and beta subunit PDH2 in the *pdh2* mutant. (E) Abundance of PsbA and other components of the PSII complex in the *Chlamydomonas* mutant lacking *CrHCF173*, the homolog of *AtHCF173*, which is necessary for PsbA translation initiation in *Arabidopsis*. The bigger dots represent other subunits of the complex of interest. See also Figure S4.

To expand the understanding of the 115 genes identified as required for photosynthesis and to elucidate the specific roles of poorly characterized genes within this set, we sought to use mass-spectrometry proteome profiling (Figure 3A) to assess the impact of the loss of each gene on the proteome. We reasoned that this would be an informative approach to characterize mutants deficient in photosynthesis because the core activities of photosynthesis are mediated by a series of highly expressed protein complexes whose abundance is affected by photosynthetic activity, regulation, and biogenesis. Indeed, many known photosynthesis-deficient mutants show differences in protein complex abundance.^32–34^ Much of the regulation of the photosynthetic apparatus is thought to occur post-transcriptionally, making protein levels a more informative readout than mRNA.^5^

When grown in light, our strains exhibit growth defects, which could confound the proteomic readout. To minimize such issues, we grew cells in the dark with acetate as carbon and energy source, taking advantage of the facts that under this condition, growth defects associated with deficient photosynthesis are eliminated, and WT cells assemble a functional photosynthetic apparatus.^16^

We obtained proteome profiles of mutants each disrupted for one of 100 genes (Figure S2A; Table S3), with at least two experimental replicates for each gene (Figures 3A and S5; STAR Methods). Our profiling dataset captured known co-depletion of proteins that form complexes such as LCIB and LCIC^35^ (Figure 3B) and known regulatory effects such as the depletion of cytochrome *b*_6_*f* in the *tca1* mutant^36,37^ (Figure 3C).

Our data also illustrated that, in most cases, *Chlamydomonas* genes behave similarly to their characterized land plant homologs. For example, based on their homology to *Arabidopsis* proteins, the algal proteins PDH2 and PDC2 are predicted to be the two subunits of pyruvate dehydrogenase E1; indeed, PDH2 and PDC2 were co-depleted in the *pdc2* mutant (Figure 3D). Another example is *CrHCF173*, a homolog of the *Arabidopsis* translation initiation factor *AtHCF173* that is required for PsbA translation initiation.^38^ As was shown for *AtHCF173*, we observed that mutation of *CrHCF173* led to the downregulation of PsbA and the entire photosystem II (PSII) complex^39,40^ (Figure 3E). The similar behavior of *Chlamydomonas* mutants compared with their land plant homologs suggests that lessons we learn in *Chlamydomonas* will also inform our understanding of photosynthesis across the green lineage.

Altogether, ~2,000 proteins were observable in most of the 100 mutant proteomes (Figure S4C; Table S5), providing extensive opportunities for analysis. Here, we focus on the major photosynthetic protein complexes.

### 23 poorly characterized genes impact biogenesis or regulation of individual chloroplast protein complexes

**Figure 4.**
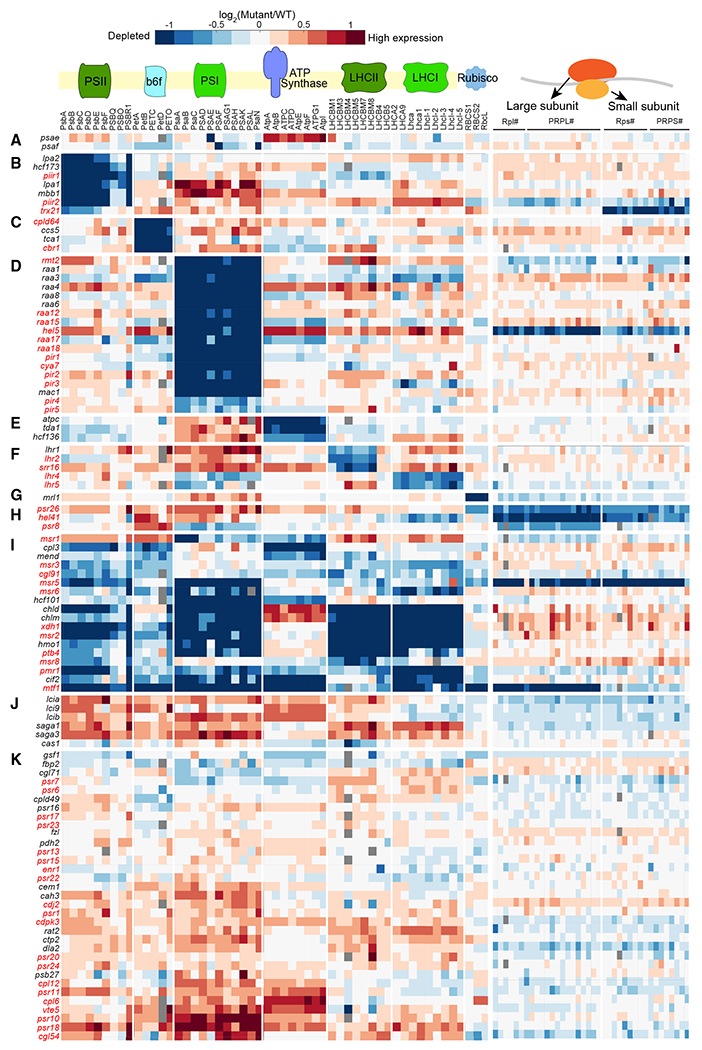
More than half of the profiled genes are required for accumulation of one or more photosynthetic complexes Relative abundances are shown for proteins (columns) in mutants (rows). Mutants labeled in red correspond to genes whose function in photosynthesis was not previously characterized. Each data point reflects the average normalized log_2_ (mutant/WT protein abundance) from two independent experiments (see Figure S4). Gray indicates that a protein’s abundance could not be measured in that mutant. (A) Mutations in the two core photosystem I proteins PSAE and PSAF have a local effect on photosystem I. (B–H) Mutants were grouped according to their impact on photosynthetic complexes: (B) photosystem II, (C) cytochrome *b*_6_*f*, (D) photosystem I, and (E) ATP synthase, (F) Light-harvesting complexes, (G) Rubisco, or (H) the chloroplast ribosomal proteins. (I) Mutants that showed depletion of multiple complexes. (J) Mutants in genes associated with the CO_2_ concentrating mechanism. (K) Mutants in other genes represented in the proteomics data. See also Figures S4 and S5.

While we observed many cases of mutants that impacted individual components of photosynthetic protein complexes, such as mutants that lack the PSI core subunits PSAE and PSAF (Figure 4A), more than half of our mutants showed proteomic defects in one or more entire complexes (Figures 4B–4I). 41 mutants led to the primary depletion of just one of the eight chloroplast protein complexes we investigated (Figures 4B–4H). These data allowed us to immediately assign roles for 23 poorly characterized genes in the biogenesis or regulation of PSII, cytochrome *b*_6_*f*, PSI, the light-harvesting complexes, or the chloroplast ribosome.

#### PSII

PSII uses light energy to extract electrons from water in the first step of the photosynthetic electron transport chain. In our dataset, mutations in seven genes led to the depletion of the entire PSII complex (Figure 4B). Three of these genes were not previously associated with PSII in any organism. One of the three, *PIIR1* (*Cre16.g658950*), encodes a protein that is predicted to localize to the chloroplast^27^ and has 6-fold higher transcript levels in light compared to dark,^41^ so it may participate in the regulation of PSII in response to light.

#### *Cytochrome* b_6_f

Cytochrome *b*_6_*f* pumps protons into the thylakoid lumen powered by photosynthetic electron flow. In our dataset, mutation of four genes led to the depletion of the entire cytochrome *b*_6_*f* complex (Figure 4C). Of these four genes, two poorly characterized ones, *CPLD64 (Cre12.g485850)*, which we validated by genetic rescue (Figure 2E; Table 1), and *CBR1* (*Cre12.g501550*), are conserved in land plants (Table S2) and were predicted to localize to the chloroplast.^27^ Given these observations, we speculate that CPLD64 and CBR1 participate in the biogenesis or stability of the cytochrome *b*_6_*f* complex.

#### PSI

PSI uses light energy to energize electrons, enabling the reduction of NADP to NADPH. In our dataset, mutations in 18 genes led to the depletion of the entire PSI complex (Figure 4D). Twelve of these genes were not previously identified as genes required for photosynthesis, including *RAA12, RAA15, RAA17-18, HEL5/CPLD46, PIR1*, and *PIR2*, which we describe in detail in later sections. Other interesting poorly characterized genes included *RMT2* (*Cre12.g524500*) and *PIR3* (*Cre01.g012200*). *RMT2* was named based on sequence homology to Rubisco large subunit N-methyltransferase (enzyme:EC:2.1.1.127), but we observed that the *rmt2* mutation did not affect Rubisco stability. Rather, it led to the depletion of PSI (Figure 4D), suggesting that RMT2 actually participates in PSI biogenesis or stability. PIR3 is conserved to land plants, has a predicted basic leucine zipper (bZIP) transcription factor domain, and is predicted to localize to the cytosol or nucleus, suggesting that it regulates the transcription of nuclear-expressed PSI genes.

#### Light-harvesting complexes

Light-harvesting complexes channel light excitation energy to the photosystems (Figure 4F). In our dataset, mutations in five genes affected the light-harvesting complexes—these genes include *LHR1* (*Cre02.g142266*), whose *Arabidopsis* homolog *CYP97A3* is required for light-harvesting complex II biogenesis,^42^ and four poorly characterized genes. Two of the poorly characterized genes, *LHR4* (*Cre01.g016350*) and *LHR5* (*Cre01.g001000*), were required for normal levels of light-harvesting complex I; whereas the other two, *SRR16* (*Cre10.g458350*) and *LHR2* (*Cre14.g616700*), affected the LHCBM proteins, the core complex of light-harvesting complex II.

#### Chloroplast ribosome

Mutations in three genes, *PSR26* (*Cre50.g761497*), *HEL41* (*Cre07.g349300*), and *PSR8* (*Cre02.g110500*), led primarily to the depletion of chloroplast ribosomal proteins (Figure 4H). The helicase HEL41 was previously found to physically associate with the chloroplast ribosomal large subunit^34^ and in our dataset had a particularly strong effect on the abundance of the large subunit, suggesting that HEL41 directly impacts ribosomal protein levels by contributing to biogenesis or stability of the large ribosomal subunit.

### 11 poorly characterized genes impact biogenesis or regulation of multiple photosynthetic complexes

Mutations in seven known and eleven poorly characterized genes led to the depletion of multiple complexes (Figure 4I). The known genes illustrate how the depletion of multiple complexes can result from different mechanisms. For example, mutants lacking chlorophyll biogenesis genes *CHLD* (*Cre05.g242000*)^43^ or *CHLM* (*Cre 12.g498550*)^44^ showed a depletion of chlorophyll-binding proteins, including both PSI and PSII complexes (Figure 4I). Other known mutants are in regulatory genes, for example, the kinase *CPL3* (*Cre03.g185200*).^25^

The poorly characterized genes affecting multiple complexes included the conserved predicted xanthine dehydrogenase/oxidase *XDH1* (*Cre12.g545101*), whose mutation led to decreased levels of PSI and PSII and their light-harvesting complexes similar to mutants in chlorophyll biosynthesis enzymes (e.g., *chld* and *chlm*). These observations suggest a role for XDH1 in pigment metabolism, possibly by preventing the activation of chlorophyll degradation by xanthine.^45^ The poorly characterized genes also included the conserved predicted chloroplast-localized protein MSR8 (*Cre09.g400312*), whose disruption impacted both PSII and light-harvesting complex II.

Disruption of the poorly characterized genes *PMR1* and *MTF1* led to the depletion of the entire photosynthetic apparatus; we discuss their characterization below.

### Characterization of factors that regulate photosynthetic apparatus biogenesis

We hypothesized that many of the poorly characterized genes encode proteins that regulate the photosynthetic machinery because many (14/24) of the known genes whose disruption led to strong depletion of the photosynthetic complexes in our proteomic experiment encode regulatory proteins (Figures 4B–4I). We focused on two subsets of the poorly characterized genes: ones whose disruption specifically impacted PSI levels and ones whose disruption had broad effects on the photosynthetic apparatus.

#### Regulators of PSI *psaA* mRNA maturation

The mRNAs encoding chloroplast-expressed proteins are constitutively expressed, and the abundance of the proteins they encode is primarily regulated post-transcriptionally.^5^ A central mechanism for this post-transcriptional regulation involves the regulators of organelle gene expression (ROGEs), nuclear-encoded factors that each promote mRNA stability/maturation (M factors) or translation (T factors) of a specific chloroplast-encoded subunit of a photosynthetic complex.^46^ In the absence of a T or M factor, the abundance of the regulated subunit drops, translation of other subunits decreases, and unassembled subunits are degraded, leading to depletion of the entire complex.^6^

We identified six known M factors among the genes required for accumulating the entire PSI complex in our proteomics (Figure 4D). One of these M factors, MAC1, is required for *psaC* mRNA stability.^47^ The other five, RAA1, RAA3, RAA4, RAA6, and RAA8, participate in the maturation of *psaA* mRNA.^48–52^

**Figure 5.**
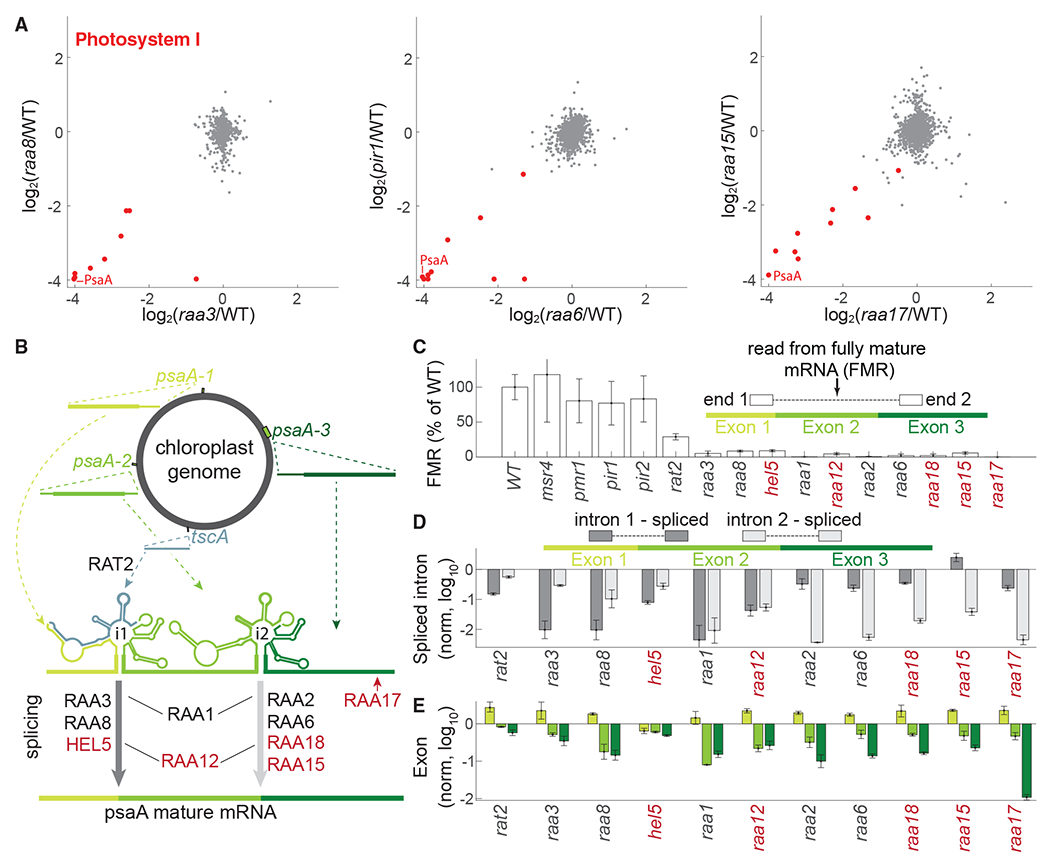
Characterization of five *psaA* mRNA maturation factors (A) Scatterplots of proteomic data of mutants of known *psaA* maturation factors (RAA8, RAA3, and RAA6) and mutants with similar proteomic profiles (PIR1, RAA15, and RAA17). For other mutants see Figure S6. (B) *psaA* mRNA maturation process. *psaA* mRNA starts as four separated RNAs expressed in the chloroplast genome, *psaA1-3* each include an exon, and *tscA* forms part of intron 1. The RNAs hybridize to form two introns that are spliced out (gray arrows) to produce the mature mRNA. This process is mediated by M factors. Known (black) and poorly characterized (red) factors from our transcriptomic dataset are shown. (C) Fully mature *psaA* mRNA levels were determined using paired-end reads. (D) *psaA* intron splicing in M factor mutants. The reads are normalized to wild type (log_10_ scale). (E) Normalized reads for each exon in the indicated mutants are depicted. Error bars represent standard error (SE). See also Figure S6

We hypothesized that other genes with similar proteomic patterns might also be M factors. We focused on seven poorly characterized genes (*HEL5, RAA17, RAA18, RAA12, RAA15, PIR1*, and *PIR2*), of which we validated three (*RAA17, RAA15*, and *PIR1*) by gene rescue (Table 1), whose mutants exhibited strong and specific depletion of the PSI complex (Figures 5A and S6). To determine whether any of these genes are M factors, we profiled the chloroplast transcriptome in mutants representing these genes and known factors (STAR Methods). Mutations in five of the poorly characterized genes, *HEL5, RAA17, RAA18, RAA12*, and *RAA15*, resulted in less than 15% of the WT levels of mature *psaA* mRNA, similar to mutants of known *psaA* mRNA maturation factors (Figures 5B and 5C), suggesting that these five genes are *psaA* M factors.

PsaA is one of the two central chloroplast-encoded components of PSI.^16^ In *Chlamydomonas*, its maturation involves a sophisticated mRNA splicing mechanism.^53^ PsaA mRNA starts as four separate transcripts that hybridize to form a structure containing two introns, which are spliced out to generate the mature mRNA (Figure 5B). This process is mediated by a ribonucleoprotein complex that includes at least 14 splicing factors.^53,54^ These splicing factors are classified based on their impact on the splicing of the two introns. By evaluating the relative splicing of each intron in the mutants using paired-end RNA sequencing (RNA-seq), we were able to classify HEL5 as impacting intron 1, RAA15 and RAA18 as impacting intron 2, and RAA12 as impacting both introns (Figure 5D). RAA17 appears to represent a new maturation factor group, which we propose directly affects exon 3 stability (Figures 5B–5E).

#### *HEL5 is required for splicing* psaA *intron 1*

*HEL5* (*Cre01.g027150*) belongs to the DEAD-box helicase superfamily (Interpro: IPR011545). Its *Arabidopsis* homolog ISE2 appears to be a general splicing factor that participates in the mRNA processing of chloroplast ribosome subunits, ATP synthase subunit AtpF, and protease ClpP1.^55^ While *Chlamydomonas* HEL5 appears to contribute to the biogenesis or stability of the chloroplast ribosome (Figure S6D), it does not affect the ATP synthase or Clp protease. Instead, we observe that the primary function of HEL5 seems to be the splicing of *psaA* intron 1 (Figures 5C and 5D), illustrating how the specificity of a splicing factor can change across evolution.

#### *RAA15 and RAA18 are required for splicing* psaA *intron 2*

In mutants lacking *RAA15* (*Cre17.g728850*) or *RAA18* (*Cre07.g351825*), we observed a 96% decrease in mature *psaA* intron 2 compared with WT, suggesting that these genes encode intron 2 splicing factors (Figures 5A, 5D, and S6). Transforming the WT allele of *RAA15* into the corresponding mutant alleviated the mutant’s growth defects to almost-WT levels (Figure 2C), providing confidence that a mutation in this gene causes the observed photosynthesis phenotype. RAA15 was previously pulled down with known intron 2 splicing factors RAA2 and RAA7,^51,56^ suggesting that these three factors function together.

#### *RAA12 is required for splicing* psaA *introns 1 and 2*

RAA12 (*Cre17.g698750*) is a member of the octotricopeptide repeat (OPR) family of regulatory RNA-binding proteins^46^ required for photosynthesis (Table S1), whose two mutant alleles showed depletion of PSI (Figures 4D and S5A). Its transcriptomic profile was similar to that of RAA1, a known M factor required for *psaA* intron 1 and 2 splicing^48^ (Figures 5D and 5E). Much like *RAA1*, we observed that *RAA12* mutation leads to the depletion of mature forms of both introns 1 and 2 (Figure 5D). Furthermore, similarly to RAA1, RAA12 was previously co-precipitated with known M factors: intron 1 splicing factors RAA4 and RAT2,^51,57^ and intron 2 splicing factor RAA7.^56^ These results suggest that RAA12 is required for the maturation of both introns.

#### *RAA17 regulates* psaA *exon 3 stability*

Transforming the WT *RAA17* (*Cre13.g566400*) allele into the *RAA17* mutant rescues the mutant’s growth to WT-like levels even under high-light conditions (Figure 2D), confirming that *RAA17* is required for photosynthesis. The *RAA17* mutant exhibits almost-complete depletion of exon 3 (<2% of WT levels), a phenotype not exhibited by any of the other mutants of known factors in our dataset, suggesting that *RAA17* is a different kind of maturation factor that specifically protects the third exon. *RAA17* is a member of the OPR family of RNA-binding proteins; thus, it is possible that it could directly bind to *psa*A. The decreased level of exon 3 is likely the cause of the decreased level of the mRNAs with spliced intron 2 observed in the *raa17* mutant. *RAA17* expression is light dependent: its expression level is 5-fold higher in light compared with dark,^41^ suggesting that it participates in *psaA* dark-to-light acclimation.

#### *RAT2 is required for* psaA *maturation but is not a limiting factor in the dark*

RAT2 is a previously known *psaA* maturation factor that participates in processing the intron 1 RNA component *tscA*^58^ (Figures 5B and S6F). As expected, a mutant strain lacking RAT2 showed photosynthetic defects in our screen, but surprisingly, it did not lead to the depletion of PSI in our protein profiling (Figure 4K). A potential explanation for this discrepancy is that the *rat2* mutant has substantially more mature *psa*A than any other maturation factor mutant in our dataset (Figures 5C–5E). This level of mature *psa*A mRNA may be sufficient for PSI production in the dark,^59,60^ conditions under which materials were collected for our proteomic analysis. Under light conditions requiring active photosynthesis, the lower levels of *psaA* mRNA would not meet the higher demand for PSI production, resulting in a photosynthesis defect.

#### psaA *mRNA maturation*

In addition to identifying and characterizing five M factors, our RNA profiling provides insights into the overall maturation process of *psaA*. In nearly all mutants that primarily impact one intron (with *raa15* being the only exception), we observed that splicing of the other intron is also impacted (Figure 5D), suggesting that each splicing site requires integrity of the other for maximal activity.

HEL5, RAA17, and RAA18 were not identified in the previous immunoprecipitation of the *psaA* mRNA maturation complex,^54^ suggesting that they are only transiently associated or act independently and demonstrating the complementary value of our approach. Together, the above findings broaden our understanding of *psaA* maturation, a key process in PSI biogenesis and regulation, and illustrate how our data can be used to rapidly functionally characterize factors with roles in photosynthesis.

### Functional specialization of chloroplast translation initiation factors

One of the most-striking observations from our data was the identification of genes whose mutants exhibited decreased levels of all four major light-reaction complexes. Two of these genes, CIF2 and MTF1, are required for chloroplast translation initiation. CIF2 (*Cre07.g341850*) likely functions as the chloroplast translation initiation factor 2 (IF2), which attaches the fMet-tRNA to the translation initiation complex, based on its homology to the characterized *Arabidopsis* IF2, *FUG1*,^61^ and CIF2’s physical interaction with the *Chlamydomonas* chloroplast ribosome.^34^

#### MTF1 is the chloroplast’s MTF and is required for translation of nearly all chloroplast-encoded proteins

**Figure 6.**
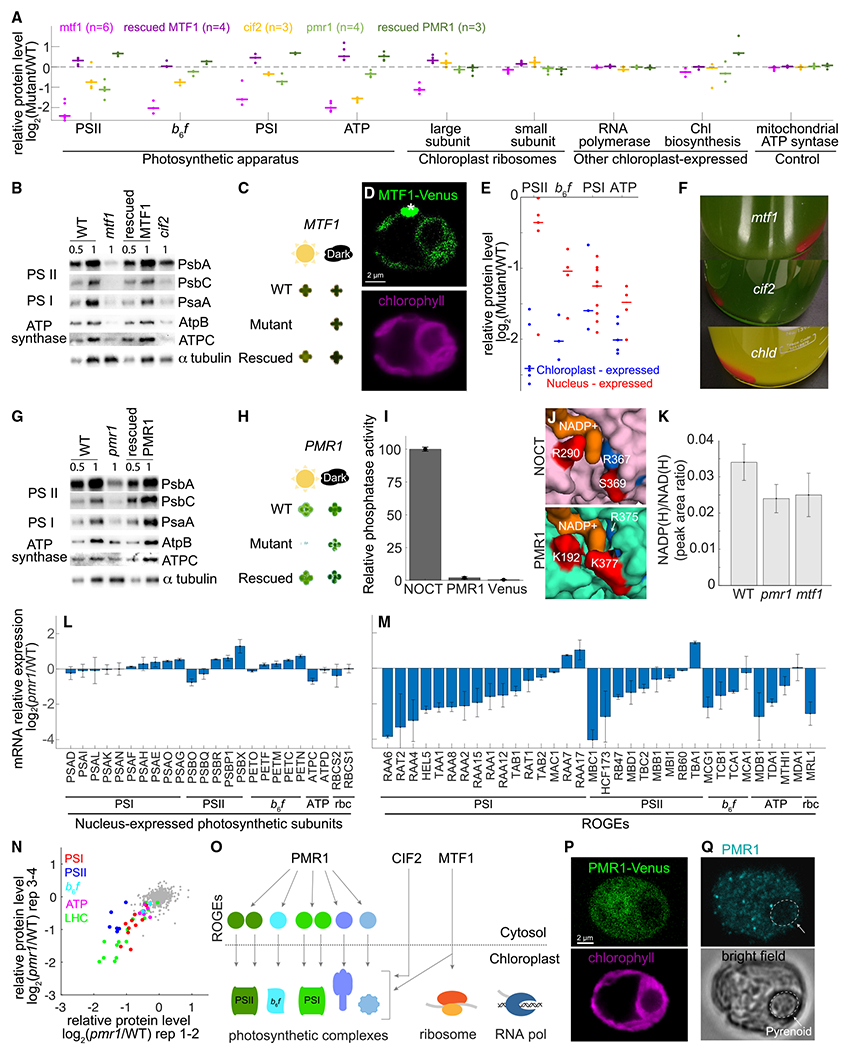
Characterization of MTF1, CIF2, and the master regulator PMR1 (A) Protein levels of chloroplast-expressed genes and mitochondrial controls. The bar represents the median of the genes in the group. See also Figures S7A–S7C. (B) Western blots showing the accumulation of photosynthetic subunits in WT, *mtf1*, rescued MTF1, and *cif2*. α-tubulin was used as a loading control. See also Figure S7D. (C) Colony growth is shown for WT, *mtf1*, and rescued *MTF1* under dark and high-light conditions. See also Figure S3. (D) Localization of Venus-tagged MTF1 (green) and chlorophyll autofluorescence (magenta). The asterisk (*) marks autofluorescence from the eyespot. (E) Comparison of chloroplast-expressed protein levels (blue) to nucleus-expressed protein levels (red) for photosynthetic complexes in the *mtf1* mutant. Each dot represents a protein. (F) Images of strains grown in tris acetate phosphate (TAP) dark. (G) Western blot for WT, *pmr1*, and rescued *PMR1* strains, as described in (B). (H) Colony growth for WT, *pmr1*, and rescued *PMR1*, as described in (C). (I) Relative NADP^+^ to NAD^+^ phosphatase activity of nocturnin (NOCT), PMR1-Venus-3xFLAG, and Venus-3xFLAG control were determined *in vitro*; see also Figure S7L. (J) The substrate-binding pocket is shown for the crystal structure of nocturnin^72^ (pink) and the AlphaFold-predicted model of PMR1 (green), with modeled NADP^+^ (orange). See Figure S7M. (K) NADP(H) and NAD(H) levels were measured using liquid chromatography-mass spectrometry (LC-MS) in WT, *mtf1*, and *pmr1* (n = 3). (L and M) mRNA levels of nucleus-expressed photosynthetic subunits (L) and ROGEs (M, Table S4) in *pmr1* relative to WT (n = 2). See also Table S5. (N) Scatterplot of *pmr1* mutant proteomic data. Each axis represents the mean of 2 experimental repeats. (O) Model. PMR1 regulates photosynthetic complexes through the ROGEs. CIF2 and MTF1 directly affect the translation of chloroplast-expressed proteins. (P) Localization of PMR1-Venus (green) and chlorophyll autofluorescence (magenta). See also Figure S7O. (Q) Indirect immunofluorescence against PMR1 in wild-type cells. Bright field is shown instead of chlorophyll because the immunofluorescence procedure washes away chlorophyll. See Figure S3F for *pmr1* control. Error bars represent SE.

*MTF1 (Cre12.g560550)* is a conserved gene whose mutant shows a severe photosynthetic phenotype. In our proteomic experiments, loss of *MTF1* expression had the strongest phenotype: the disruption of this gene resulted in the depletion of the entire photosynthetic apparatus and most of the chloroplast-expressed proteins (Figures 6A, 6B, and S7A). We validated this phenotype by genetic rescue, which alleviated the observed growth defect in the mutant to nearly WT growth under high-light conditions (Figure 6C), and recovered expression of chloroplast-expressed proteins (Figures 6A and 6B).

MTF1 was previously annotated as a putative MTF based on sequence similarity to known enzymes. MTF generate fMet-tRNA, which is the tRNA needed for translation initiation in bacteria.^62^ In contrast to bacteria, eukaryotes do not use fMet-tRNA for cytosolic translation, but the chloroplast and mitochondria within eukaryotic cells require this tRNA for translation initiation. Indeed, we found that MTF1 has a similar AlphaFold-predicted structure to the known *E. coli* enzyme MTF, with the active-site key residues and hydrophobic pocket conserved^63,64^ (Figures S7E and S7F). These similarities validate the annotation of MTF1 as a MTF.

In theory, MTF1 could provide fMet-tRNA for the chloroplast or the mitochondria. We found that Venus-tagged MTF1 localized exclusively to the chloroplast (Figure 6D). The strong effect of *mtf1* mutants on chloroplast-expressed proteins and not on mitochondrial-expressed proteins (Figure 6A) also suggest that it is primarily active in the chloroplast. Consistent with the idea that MTF1 primarily affects chloroplast-encoded photosynthetic subunits, we observed that in the *mtf1* mutant, chloroplast-expressed subunits tended to be more depleted than their nuclear-expressed counterparts (Figure 6E), suggesting that the depletion of the nuclear-expressed subunits was a secondary effect due to degradation of incompletely assembled complexes. Together, our results strongly suggest that MTF1 is the MTF that mediates chloroplast translation initiation.

#### Translation initiation factors MTF1 and CIF2 are dispensable for normal levels of several chloroplast-expressed proteins

If all chloroplast-expressed proteins required formylmethionine-tRNA as is thought to be the case for *E. coli* proteins,^65^ we would have expected that MTF1 and CIF2 would be required for translation of all chloroplast-expressed proteins. Surprisingly, we found that *mtf1* and *cif2* mutations did not affect levels of the two chloroplast-expressed proteins required for chlorophyll biosynthesis in the dark, chlB and chlL (Figures 6A, S7A, and S7B). Consistent with this observation, *mtf1* and *cif2* mutants were green when grown in the dark (Figure 6F), whereas strains without the chlB/L/N complex are yellow in the dark.^66^ *mtf1* and *cif2* mutants also did not show downregulation of chloroplast-expressed RNA polymerase (*Rpo* genes, including the essential protein rpoA^67^) (Figures 6A, S7A, and S7B). These observations suggest that translation of certain subsets of chloroplast-expressed proteins can use non-canonical translation initiation mechanisms that do not require formylmethionine-tRNA.

### PMR1 is a master regulator of photosynthesis

Our data suggest that the poorly characterized protein PMR1 (*Cre10.g448950*) coordinates the expression of multiple photosynthetic complexes by acting at the level of nuclear gene expression control. *pmr1* mutants showed severe photosynthetic growth deficiency and depletion of light-reaction complexes (most significantly PSI and PSII, and light-harvesting complex I), (Figures 4I, 6A, 6G, and 6N). These defects were all rescued by transforming the mutant strain with the WT allele (Figures 6A, 6G–6H, and S7A). Consistent with a regulatory role of PMR1, expression of the WT PMR1 allele under the strong PSAD promotor in the rescued strain led to the overexpression of most of the photosynthetic complexes (Figures 6A, 6G, and S7H).

PMR1 is a member of the CCR4-NOT family and shows the highest sequence homology (Table S6) and a similar predicted structure (Figure S7I) to nocturnin (NOCT) (Kyoto encyclopedia of genes and genomes [KEGG]: K18764), a protein that has been identified as a circadian-controlled master regulator that affects metabolism and hundreds of transcripts in animals.^68–70^ Consistent with NOCT-like characteristics, we observed that PMR1 has periodic expression^71^ (Figure S7J), and the disruption of its expression influences the levels of hundreds of mRNAs (Figure S7K).

Recent work showed that human and fly NOCT act as phosphatases that convert NADP(H) to NAD(H),^72^ which then has secondary effects on the transcriptome. We sought to determine using an *in vitro* assay whether PMR1 also acts as an NADP(H) phosphatase but observed only very minor activity (50-fold lower than NOCT) (Figures 6I and S7L). We further analyzed the predicted binding pocket for the adenine in NADP^+^ in PMR1 by structural predication, as compared with that of NOCT, and identified two residues that are different in PMR1: K192 and K377 in PMR1, corresponding to R290 and S369 in NOCT. K192 could disrupt the binding of NADP(H) and K377 may partially block the binding pocket, decreasing enzymatic activity on NADP(H) (Figures 6J and S7M). Finally, an NADP(H) phosphatase mutant would be expected to show an increase in the ratio of NADP(H) to NAD(H),^72^ but the *pmr1* mutant did not show an increase in this ratio (Figure 6K). Together, these results suggest that PMR1’s primary activity is not as an NADP(H) phosphatase; instead, PMR1 may directly regulate mRNA levels, similar to the rest of the characterized members of the CCR4-NOT family.^73,74^

Our RNA-seq analysis suggests that PMR1 regulates the levels of photosynthetic complexes through broad control of the ROGEs, nuclear-encoded factors that each regulate the mRNA stability or translation of one or two chloroplast-expressed genes.^46^ The *pmr1* mutant did not show significant depletion of mRNAs encoding nuclear-encoded subunits of photosynthetic complexes (Figure 6L). Instead, the *pmr1* mutant exhibited strong depletion of ~20 ROGEs that together regulate all major photosynthetic complexes, most notably ROGEs required for biogenesis of PSI and PSII (Figure 6M; p < 0.0016, Mann-Whitney U test comparing the ROGE mRNA distribution to the distribution of all measured mRNAs). Since the depletion of even one ROGE can lead to the depletion of an entire photosynthetic complex, we propose that this downregulation of ROGEs explains the observed broad and specific (Figures 6A, 6N, and S7N) downregulation of all photosynthetic complexes in the *pmr1* mutant (Figure 6O).

If PMR1 directly regulates the mRNA of nuclear-expressed genes, we would expect it to localize to the cytosol and/or nucleus. Consistent with this, fluorescently tagged PMR1 localized to the cytosol and nucleoplasm (Figures 6P and 6Q). Intriguingly, a substantial fraction of the protein also localizes to the chloroplast. This additional site of localization suggests the possibility that PMR1 participates in retrograde regulation—signaling from the chloroplast to the nucleus and cytosol to regulate nuclear-expressed genes.^75^

## DISCUSSION

In this study, we identified with high confidence (FDR < 0.11) 115 genes required for photosynthesis, including 70 whose functions in photosynthesis had not been previously characterized in any organism. We then showed that mutant proteomes provide key insights into the functions of these genes in photosynthesis, in many cases allowing the assignment of genes to specific pathways.

We identified five ROGEs that are essential for the biogenesis of PSI. Including these genes, 76% (16/21) of genes with known functions in our dataset that lead to the depletion of an entire photosystem complex are ROGEs (Figure 4), demonstrating their significant impact on photosynthesis.

Growing evidence indicates that ROGEs play a regulatory role rather than being merely required for complex biogenesis^46^: different ROGEs affect different chloroplast-encoded genes,^5^ are differentially transcriptionally regulated,^59^ and participate in feedback loops,^6,76^ a classical transcription network motif.^77^ Moreover, several ROGEs can coregulate the same protein^56,76^ (Table S4), and the expression of photosystem proteins with a stronger effect on growth, including the largest subunit of each complex, tends to be impacted by more ROGEs (Table S4). Our results further support a regulatory role for ROGEs by showing that different ROGEs can be limiting factors in different conditions: RAT2 is a limiting factor for *psaA* expression in the light but not in the dark (Figures 4K and 5C–5E), and by discovering that multiple ROGEs are controlled by a master regulator (Figure 6O). Together, ROGE-mediated regulation raises the intriguing possibility that during the endosymbiosis process, as transcriptional regulation in the chloroplast was lost,^5^ ROGEs evolved to replace transcription factors in a regulatory network for chloroplast-expressed proteins.

In order to respond effectively to changing conditions, the cell must simultaneously regulate multiple photosynthetic complexes. Such coordinated regulation cannot be achieved by the ROGEs alone, since each regulates only one or two chloroplast-encoded proteins.^5^ Our results suggest the existence of two mechanisms that operate on a larger scale to coordinate the expression of multiple complexes.

First, the cell appears to leverage the chloroplast translation machinery to coregulate multiple complexes. Specifically, while translation factors MTF1 and CIF2 may look like classical housekeeping genes, our data suggest that they are leveraged for regulatory functions. Whereas classical housekeeping translation initiation factors mediate all translation,^78^ MTF1 and CIF2 each affect specific subsets of chloroplast-expressed proteins, a property associated with regulatory factors.^79^ CIF2 is mostly required for expression of photosynthetic machinery, whereas MTF1 loss also affects ribosomal large subunits (Figures 6A and S7A–S7C). Consistent with a regulatory role, MTF1 overexpression leads to overexpression of proteins downregulated in the *mtf1* mutant (Figure 6A). The differences in the proteomic impacts of *mtf1* and *cif2*, combined with the differential regulation of the *MTF1* and *CIF2* transcripts (Figure S7G), suggest that MTF1 and CIF2 coordinate chloroplast gene expression in response to light and nitrogen availability.

Second, our data suggest that the master regulator PMR1 regulates the mRNA levels of multiple nuclear-encoded ROGEs, thus coordinating the expression of the overall photosynthetic apparatus. We hypothesize that the higher-level regulatory mechanisms mediated by PMR1, MTF1, and CIF2 are essential for the cell’s rapid and coordinated response to changes in growth conditions.

More than 65% of the 115 genes we identified as required for photosynthesis have homologs in land plants (Figure S1H). In most cases, the functions of these conserved genes appear to be similar in *Chlamydomonas* and land plants, supporting the value of *Chlamydomonas* as a model system and expanding the significance of our findings. Genes with no clear homologs in land plants could reflect homolog search failure due to sequence divergence^80,81^ and/or different evolutionary innovations in the algal lineage such as the algal-specific CO_2_-concentrating mechanism (CCM), the study of which has the potential to enhance crop yields.^82^ We anticipate that future studies of the genes identified here and explored in our proteomics dataset will enable further discoveries in photosynthesis.

### Limitations of the study

Considering our FDR cutoff of 0.11, up to 11% of our hits may be false positives. We have validated by genetic rescue 12 of the 70 genes not previously known to be required for photosynthesis; future work on other genes will require independent validation. In addition, although protein localization by Venus-tagging is generally reliable,^30,31^ increased confidence in the conclusions on cellular localization will require validation by an independent method such as immunofluorescence.^31^ While we have initiated here the characterization of several of the identified genes, additional work is needed to fully characterize the molecular mechanisms by which they and other factors impact photosynthesis.

## STAR★METHODS

### RESOURCE AVAILABILITY

#### Lead contact

Further information and requests for resources and reagents should be directed to and will be fulfilled by the lead contact, Martin C. Jonikas (mjonikas@princeton.edu).

#### Materials availability

All unique/stable reagents generated in this study are available from the lead contact upon request.

#### Data and code availability

- Data have been deposited and are publicly available:
  - The raw RNA and DNA sequencing data are available in NCBI with accession ID SRP441891: https://www.ncbi.nlm.nih.gov/Traces/study/?acc=SRP441891&o=acc_s%3Aa
  - The raw proteomic data are available in ProteomeXchange Consortium via the PRIDE partner repository with dataset identifier PXD036908: http://www.ebi.ac.uk/pride/archive/projects/PXD036908
- This paper does not report original code.
- Any additional information required to reanalyze the data reported in this paper is available from the lead contact upon request.

### EXPERIMENTAL MODEL AND SUBJECT DETAILS

#### Strains and culture conditions

We performed all experiments on Tris Acetate Phosphate (TAP) TAP or Tris Phosphate (TP) media with revised trace elements.^88^ TP media had the same recipe as TAP, but the acetic acid was omitted and HCl was added instead to adjust the pH to 7.5. We propagated strains robotically on TAP agar as previously described.^29^

All mutants used in this study were from the C.^89^ PhotosyntheLiP library.^25^ We used the library’s parental strain, CC-4533, as wild type. We backcrossed mutants to a CC-1690 mt+ transformant carrying a hygromycin resistance cassette (WT-hyg), which has high mating efficiency with the CLiP strains.

We performed spot tests and backcrossing with a subset of 1,781 out of the 3,109 mutants deficient in photosynthetic growth identified previously.^25^ This subset had been propagated in the laboratory as colony arrays in 96-colony format since the library’s original construction; whereas propagation of the remaining strains had stopped by the time this study began.

We focused our efforts on characterizing insertions with mapping confidence levels^25^ of 1-3.^25^ The 1,781 mutants carried insertions into 1,616 genes mapped with confidence levels 1-3.

### METHOD DETAILS

#### Automated spot tests

We used a RoToR robot (Singer) to replicate colony arrays in 384-colony format from the TAP agar plates on which the 1,781 mutants were propagated onto three agar plates: one TAP, and two TP. We grew the TAP plate in the dark for about a week before imaging; and we acclimated the two TP plates overnight at ~100 μE/m^2^/s, and then moved them to high light ~750 μE/m^2^/s for 2-3 days before imaging (using Lumigrow Lumibar lights, catalog number 8100-5502; equal levels of red, blue, and white light). We photographed the plates using a PhenoBooth imager (Singer). We performed the experiment in four replicates: two independent experiments with a technical replicate in each experiment.

To calculate the “normalized colony photosynthetic growth” we analyzed the pictures using MATLAB. We selected parameters and the algorithm to match as closely as possible our observations by eye. We used a MATLAB script to identify and remove the background and to calculate colony size, which we determined based on the number of green pixels. We further added a 0.5-1 adjustment based on how dark green the pixels are, because when colonies are more dense, they become a darker green. We limited the effect of the color to 0.5-1 to put more emphasis on the colony’s actual size, which we felt more closely reflects the colony’s growth. This normalization is done automatically using MATLAB based on the color levels, and all the values are relative. We normalized the colony size in each plate by the median size of the 10 largest colonies. We then normalized the size of each colony on the high light plates by the size of the corresponding colony on the corresponding TAP dark plate. We performed the second normalization to rule out mutants with a slow growth phenotype that is not specific to photosynthesis.

#### Pooled backcrossing

We performed initial backcrossing experiments with two subsets of mutants labeled MK (26 plates) and AB (10 plates), which together contained the 1,781 mutants, with some mutants being present in both subsets. After obtaining initial results with these subsets, we re-arrayed the most promising mutants in 96-colony format onto four plates labeled NP. The NP plates included 1) mutants containing insertions linked to photosynthetic defects in the initial backcrosses, 2) insertions in genes that were identified as high-confidence hits in our previous study,^25^ and 3) mutants that were yellow or brown. Additionally, to check the method’s replicability, we generated a control plate which contained mutations in genes that were not hits and carried insertions whose disruption likely did not result in a photosynthesis defect. The genes disrupted in mutants on the control plate included 1) known flagellar genes and 2) genes that were represented by more than 35 barcodes, no more than 2 of which were hits in our original pooled photosynthesis screen^25^ (in other words, many mutants were available for these genes and the vast majority of these mutants did not exhibit a photosynthesis defect). Using the NP and control plates, we performed a final backcrossing experiment that included two biological repeats of the NP plates and one biological repeat of the control plate.

The backcrossing approach was adapted from the pooled mating (Multiplexed Bulked-Segregant Pool) protocol described previously.^26^ Our protocol is illustrated in Figure S1. Each experimental replicate consisted of the following steps:

1. Mating: We scraped and pooled mt- mutant strains from 96-colony format arrays into flasks containing low-nitrogen gamete-induction medium.^26^ We pooled 60-150 colonies into each 250 ml flask containing 50 ml of gamete-induction medium. We resuspended a similar quantity of WT-hyg into separate flasks containing the same media. We used a cell counter to verify that the strains and the WT-hyg cells were at a similar concentration. We shook flasks at 90 RPM for 5-7h in low light (~40 μE) for mating induction. Then for each flask of mutant strains, we mixed 700ul of mutant strains (mt-) and 700ul of WT-hyg in a 1.5 ml Eppendorf tube, incubated them at low light (~40 μE) without shaking for one hour, then gently spread them on two TAP agar plates. We incubated the plates overnight in very low light (~30 μE). In the morning, we wrapped the plates in aluminum foil and kept them in the dark for 7 days.
2. Meiosis: We removed most of the unmated cells by scraping the agar surface using a sharp razor, and moved the plates to low light (~30 μE) for meiosis induction and initial proliferation for ~5 days. We used a light microscope to check the sporulation efficiency.^90^ We pooled the strains into liquid media (TP) for competitive growth.
3. Light and cassette selections (competitive growth): We added hygromycin to our media to ensure that only backcrossed strains were measured. The mutant library does not have hygromycin resistance, so the original CLiP mutants cannot grow on this media. The WT-hyg strain has hygromycin resistance but does not have barcodes, so it will not affect the barcode counting. We inoculated pooled strains at ~2 × 10^4^ cells mr^−1^ into TAP + hygromycin (15 μg/ml) 1L bottles for dark growth (3 replicates) and TP + hygromycin (15 μg/ml) 1L bottles for high light growth (3 replicates; except of the 1^st^ experiment where we also included hygromycin (15 μg/ml) + paromomycin (5 μg/ml) conditions). We bubbled air into the bottles and stirred them using magnetic stirrers at 200 rpm. We exposed the TP cultures to 100 μE for overnight light acclimation, then to 750 μE for the remainder of the growth (using Lumigrow Lumibar lights, catalog number 8100-5502; equal levels of red, blue, and white light). When the cells reached a concentration of approximately 2 × 10^6^ cells ml^−1^, we harvested 10^8^ cells for DNA extraction by centrifugation and flash-freezing the pellet in liquid nitrogen.

**Table:** 

Name	Plates in backcrossing	Competition experiments
Exp1 (AB replicate 1)	AB set (10 plates)	2 TAP Hygromycin dark and 2 TP Hygromycin light; 1 TAP hygromycin + paromomycin dark and 2 TP hygromycin + paromomycin light
Exp2A (MK 1-12 replicate 1)	1^st^ half of MK set (12 plates)	3 TAP hygromycin dark and 3 TP hygromycin light
Exp2B (MK 13-26 replicate 1)	2^nd^ half of MK set (14 plates)	3 TAP hygromycin dark and 3 TP hygromycin light
Exp3A (MK 1-12 replicate 2)	1^st^ half of MK set (12 plates)	3 TAP hygromycin dark and 3 TP hygromycin light
Exp3B (MK 13-26 replicate 2)	2^nd^ half of MK set (14 plates)	3 TAP Hygromycin dark and 3 TP Hygromycin light
Exp4 (AB replicate 2)	AB set (10 plates) + 3 plates from MK set.	3 TAP hygromycin dark and 3 TP hygromycin light
NP plates	2 biological replicates of NP set (4 plates) + 1 biological repeats of control set (1 plate).	For each biological replicate: 3 TAP hygromycin dark and 3 TP hygromycin light

Next, we extracted the DNA and prepared the barcode libraries as described,^24^ and sent the libraries for Illumina sequencing at the Princeton Genomics Core Facility.

After demultiplexing, the barcodes where quantified, normalized, and used to calculate the growth score as described in “barcode quantification, normalization, and growth score calculation” in the “quantification and statistical analysis” section below.

### Validating insertion sites by PCR

We adapted the check PCR protocol from the CLiP website (https://www.chlamylibrary.org/about), where we used the G1 and G2 primers to validate the existence of the expected insertion (Figure S2). We used the primers suggested for each strain on the CLiP website. We considered the mapping validated if we got a larger PCR product for the mutant than for the wild type, or if we obtained a PCR product for the wild type and not for the mutant in at least 2 experiments (Figure S2).

### Validating insertion sites by DNA sequencing

The strains were grown in the dark condition, and the DNA was extracted using the same method as above. The DNAs were sent to Princeton Genomics Core Facility for library preparation and whole genome sequencing.

The paired-end 150nt reads were aligned to a reference file that combined the v5.5 Chlamydomonas genome (from Phytozome), the chloroplast and mitochondrial genomes (from NCBI: chloroplast_BK000554.2.gb and mitochondrion_U03843.1.gb) and our CIB1 cassette,^25^ using the command “bowtie2 -sensitive-local -k 10 -I 100 -X 650 -S”. The resulting SAM files were filtered to extract only read pairs indicating insertion junctions (where the primary alignment was discordant with one side aligning to the CIB1 cassette and the other side aligning to the genome). The resulting genomic positions corresponding to likely cassette insertion positions were clustered (using scipy.cluster.hierarchy.fclusterdata(t=3000, criterion=’distance’, method=’average’)). For each mutant, all clusters containing 4 or more reads were plotted to show the detailed read locations and orientations, as well as the putative insertion positions according to the original library data.^25^

Additionally, for each such plot, the concordant read pairs spanning each genomic position were counted and plotted. The resulting plots were evaluated manually to determine the most likely insertion position(s), based on the numbers of matching reads, whether the reads originated from both sides of the insertion position (much less likely for junk fragments), and whether there were concordant read pairs spanning the position (real cassette insertions should not have concordant read pairs spanning them, since the cassette is much longer than the sequenced fragment size).

### Selection of 115 high-confidence hits

In our experiment, 148 mutants in 136 genes showed normalized light growth after backcrossing that fell below the 0.34 threshold (FDR = 0.1).

First, we validated that the insertions were mapped to the correct genes. We validated the mapping for 117/136 of those genes (86%) by PCR and DNA sequencing (Figures 1F and S2; Table S1). The 19 unvalidated genes were removed from the list.

Next, we removed some of the hits to improve the quality of the data set as described below:

1. Six genes (*Cre06.g262900, Cre03.g158950, Cre12.g521450, Cre13.g578600, Cre17.g728700, Cre02.g106950*) were represented by only one mutation that was in a strain that also included a mutation in an established photosynthetic gene or in a gene with multiple hits in our data set. In these cases, we assumed that the phenotype originated from the well-established gene and removed the 2^nd^ gene from the hit list.
2. Five strains had two hits in each (LMJ.RY0402.172741: Cre13.g584250 + Cre12.g554400, LMJ.RY0402.187220: Cre11.g481115 + Cre07.g326010, LMJ.RY0402.210483: Cre10.g458700 + Cre03.g211185, LMJ.RY0402.166642: Cre03.g155001 + Cre16.g660390 & Cre16.g660430, LMJ.RY0402.176469: Cre06.g296500 & Cre06.g296550 + Cre16.g687294 & Cre16.g687406). While both genes may be required for the photosynthetic growth, it is more probable that one is the real hit and the other is piggybacking on its phenotype. Hence, we counted them as one and concentrated on the one more likely to be connected to photosynthesis (Cre13.g584250, Cre11.g481115, Cre10.g458700, Cre03.g155001, Cre16.g687294). In Table S1, we state the reason for the choice and mention that the effect can be from the other gene.
3. We removed *Cre09.g407650* from the gene hits list because we observed in the proteomic data that *Cre09.g407650* is not downregulated in the corresponding mutant (Figure S5C). The insertion in that mutant was in the 3’ UTR, consistent with a mild effect on protein levels.

We then added 10 genes as described below:

In our statistical analysis, we looked at genes with insertion mapping confidence levels of 1–3 and excluded confidence level 4 insertions because only 58% of these mutants are correctly mapped.^25^ However, there were 3 cases where we did validate the insertion of confidence level 4 hits (LMJ.RY0402.124891: *Cre16.g665750*, LMJ.RY0402.207089: *Cre01.g040050*, LMJ.RY0402.097626: *Cre12.g501550*), so we added those three genes to the hit list.

Last, we added 7 genes based on manual analysis of the data (LMJ.RY0402.176891: *Cre01.g022681*, LMJ.RY0402.119871: *Cre06.g273700*, LMJ.RY0402.091258: *Cre09.g415500*, LMJ.RY0402.174216: *Cre09.g415700*, LMJ.RY0402.049481: *Cre02.g091750*, LMJ.RY0402.049829: *Cre11.g467573*, LMJ.RY0402.208107: *Cre16.g668700*). In most of these cases, the gene was not a hit in the original analysis because it was not a hit in one replicate, but the replicate is not reliable due to an obvious reason such as very low reads. After removing a problematic experiment, the gene is a hit. In Table S1, we mention in each of these cases why the gene was included in the hit list.

After these edits, our list contained 115 high-confidence genes.

### Comparison to hits from previous large-scale studies

We compared our 155 high-confidence genes to two sets of hits: 1) previously-identified high-confidence hits, and 2) previously low-confidence hits; which we obtained from three previous large-scale studies.^21,24,25^

**Previously-identified high-confidence hits** consisted of high-confidence hits from Li et al.^25^ and genes in the photosynthesis clusters in Fauser et al.^24^ Fauser et al. clustered mutants together based on their phenotype in over 100 different conditions. The work identified two clusters of genes relevant to photosynthesis. The first cluster is the light-sensitive group, where all the hits are relevant to our study; the second cluster is the photoautotrophic light-insensitive. In this second cluster, the clustering is based on phenotypes across many conditions; however, the only condition similar to our experiments is Photoautotrophic 1–3, so we took only the genes whose mutants exhibited pronounced phenotype in this condition: *Cre14.g616600, Cre01.g016514, Cre03.g194200, Cre03.g188700, Cre10.g423500, Cre06.g259100, Cre11.g467712*. We merged the hits from Li and Fauser. This procedure yielded 51 high confidence hits, of which 41 were also high-confidence hits in our study.

**Previously low-confidence hits** consisted of a subset of the 260 low-confidence hits from Li et al.^28^ and the 253 low-confidence hits from Fauser et al.^24^ that were represented in the collection of mutants we analyzed. Neither data set had FDR calculations. While both datasets include genes truly required for photosynthesis, methodological limitations of the studies mean that these datasets also include a substantial number of false positives, making validation by our orthogonal method valuable. In low-confidence hits from Li et al., many of the genes are represented by only one mutant, and others are represented by several mutants but only a small fraction of these mutants shows a photosynthetic phenotype. So, there is a high chance that the photosynthetic phenotype comes from a second-site mutation. In the Wakao study, the authors showed that in most cases their insertion is linked to the photosynthetic phenotype; however, their insertions typically were associated with large deletions that affected several genes. Wakao et al. chose to assign the phenotype to one of the disrupted genes in each of the mutants, primarily based on the literature. Although this connection is often correct, it does not have an experimental/statistical basis.

To create the low-confidence data sets, we first merged the Li and Wakao datasets with 260 and 253 hits respectively. We then took the subset of this merged list of genes that overlaps with the ~1,616 genes that were included in our initial data set. If a gene was also in the previously-identified high-confidence hits, it was removed from this list. This procedure yielded 219 previously low-confidence hits, of which 32 were high-confidence hits in our study.

### Mutant gene rescue protocol

The plasmids for complementation were generated as described previously.^31^ 4 of the 16 plasmids were based on the pLM005 backbone, and the remaining 12 were based on the pRAM118 plasmid where the paromomycin resistance cassette was replaced with a hygromycin resistance cassette.^86^ All plasmids expressed the gene of interest from a PSAD promoter and appended a Venus-3xFLAG tag to the protein sequence.

In the gene rescue protocol, we transformed mutant cells with the linearized plasmid expressing the gene disrupted in the mutant. The linearization and transformation process was carried out as previousl, until the selection, which was carried out as follows. For plasmids with hygromycin resistance cassette, we used hygromycin-based selection. The cells were plated on 1.5% agar TAP plates with hygromycin (20 μg/ml) and paromomycin (μg/ml) and placed under very dim light for five days, then transferred to light (~100 μE) for 1–2 weeks until colonies of a sufficient size for picking appeared. For plasmids with paromomycin resistance cassette, we could not use drug selection because CLiP strains already have paromomycin resistance, so we used light selection instead. This selection could be used only for mutants that grow poorly under light conditions. For each of these strains, we included a control where we transformed the mutant with a different plasmid of similar size to determine if transformation with any plasmid could reverse the phenotype, e.g., by creating a second-site suppressor mutation. We only considered a rescue successful when the transformation of the correct gene led to growth under light conditions and the control transformation did not. We plated the cells on 1.5% agar TP plates with paromomycin (20 μg/ml). We gradually increased the light intensity to allow for light acclimation. We left the plate on the shelf overnight for five days under 30 μE, three days under ~100 μE, and finally 3-4 days under ~600-700 μE light.

Next, we validated the rescues by robotic spot tests. After the rescue, we picked ~40 transformants from each rescued mutant to check their photosynthetic phenotype. We used RoToR robot (Singer) to replicate each plate with transformants, wild type and mutants to TP and TAP plates, in order to check their growth under TP highlight (550-1100μE) compared to their growth under TAP dark conditions. Then we took 2-4 promising colonies (3 replicates for each) into the plate with wild type and the original mutants (RP 1-4 plates). We used those plates to validate our rescued phenotype. We have at least two independent experiments for each RP plate.

Gene rescue is notoriously challenging in Chlamydomonas due to difficulties with PCR amplification and expression of heterologous genes,^28–30^ so we performed this part as a “screen”. We used plasmids with the 36 genes we managed to clone (Cre01.g014000, Cre01.g015500, Cre01.g016350, Cre01.g022681, Cre01.g040050, Cre02.g073850, Cre02.g106950, Cre02.g142266, Cre03.g158950, Cre03.g188700, Cre05.g243800, Cre05.g248600, Cre06.g258566, Cre06.g262900, Cre06.g279500, Cre07.g350700, Cre09.g396920, Cre10.g420561, Cre10.g433400, Cre10.g448950, Cre10.g466500, Cre11.g467682, Cre12.g485850, Cre12.g498550, Cre12.g521450, Cre12.g524250, Cre13.g566400, Cre13.g578650, Cre13.g584250, Cre13.g608000, Cre16.g658950, Cre16.g675246, Cre17.g728850, Cre12.g560550, Cre09.g396250, Cre16.g687294), to try to rescue its mutant strain once, and continued with the strains that we managed to rescue. Our success rate of ~44% is close to the maximum expected even if all were real hits, considering that only 30%–50% of transformed constructs express in medium-throughput efforts.^31^ Many of the failed rescues are likely due to challenges with transformation into Chlamydomonas,^28–31^ detrimental effects of the GFP tag or the constitutive promoter with some of the genes, and the inherent limitations of our approach, including that rescue of each mutant was only attempted once.

The rescued mutants generated in this study are listed below:

1. Rescued CHLM was generated by transforming plasmid A134 (hygromycin resistance) into the CLiP mutant LMJ.RY0402.228123, which carries a disruption in the *Cre12.g498550* gene.
2. Rescued PSR1 was generated by transforming plasmid *pRAM+49;50* (hygromycin resistance) into the CLiP mutant LMJ.RY0402.077016, which carries a disruption in the *Cre10.g433400* gene.
3. Rescued CPL6 was generated by transforming plasmid A249 (hygromycin resistance) into the CLiP mutant LMJ.RY0402.046095, which carries a disruption in the *Cre06.g279500* gene.
4. Rescued CPL12 was generated by transforming plasmid A253-2 (hygromycin resistance) into the CLiP mutant LMJ.RY0402.180319, which carries a disruption in the *Cre10.g466500* gene.
5. Rescued CGL54 was generated by transforming hygromycin resistance plasmid (N/A) into the CLiP mutant LMJ.RY0402.057931, which carries a disruption in the *Cre02.g073850* gene.
6. Rescued TPK1 was generated by transforming plasmid pRAM+77;78 (hygromycin resistance) into the CLiP mutant LMJ.RY0402.207089, which carries a disruption in the *Cre01.g040050* gene.
7. Rescued PSR5 was generated by transforming plasmid pRAM+69;70 (hygromycin resistance) into the CLiP mutant LMJ.RY0402.176891, which carries a disruption in the *Cre01.g022681* gene.
8. Rescued CPLD64 was generated by transforming plasmid A258 (hygromycin resistance) into the CLiP mutant LMJ.RY0402.234057, which carries a disruption in the *Cre12.g485850* gene.
9. Rescued TBA2 was generated by transforming plasmid pRAM+103;104 hygromycin resistance) into the CLiP mutant LMJ.RY0402.164167, which carries a disruption in the *Cre13.g578650* gene.
10. Rescued PIR1 was generated by transforming plasmid A202 (hygromycin resistance) into the CLiP mutant LMJ.RY0402.044496, which carries a disruption in the *Cre01.g014000* gene.
11. Rescued PMR1 was generated by transforming plasmid B451 (hygromycin resistance) into the CLiP mutant LMJ.RY0402.248779, which carries a disruption in the *Cre10.g448950* gene.
12. Rescued MTF1 was generated by transforming plasmid M1A (hygromycin resistance) into the CLiP mutant LMJ.RY0402.193706, which carries a disruption in the *Cre12.g560550* gene.
13. Rescued PBS27 was generated by transforming plasmid Y7 (paromomycin resistance) into the CLiP mutant LMJ.RY0402.255772, which carries a disruption in the *Cre05.g243800* gene.
14. Rescued RAA6 was generated by transforming plasmid T675 (paromomycin resistance) into the CLiP mutant LMJ.RY0402.208103, which carries a disruption in the *Cre07.g350700* gene.
15. Rescued RAA5 was generated by transforming plasmid T666 (paromomycin resistance) into the CLiP mutant LMJ.RY0402.254076, which carries a disruption in the *Cre17.g728850* gene.
16. Rescued RAA17 was generated by transforming plasmid J6/T791 (paromomycin resistance) into the CLiP mutant “LMJ.RY0402.133008, which carries a disruption in the *Cre13.g566400* gene.

#### Confocal microscopy

We performed confocal imaging as described previously.^31^ Colonies were transferred to a 96-well microtiter plate with 100 μL TP liquid medium in each well and then pre-cultured in air under 150 μmol photons m^−2^ s^−1^ on an orbital shaker. After ~16 hr of growth, 10 μL cells were transferred onto an μ-Slide 8-well glass-bottom plate (Ibidi) and 200 μL of 1% TP low-melting-point agarose at ~35 °C was overlaid to restrict cell movement. Cell samples were imaged using a Leica SP5 confocal microscope with the following settings: Venus, 514 nm excitation with 530/10 nm emission; and chlorophyll, 514 nm excitation with 685/40 nm emission. All confocal microscopy images were analyzed using Fiji.^87^ For each strain, a confocal section through a cell showing the predominant localization pattern was captured and analyzed.

#### Proteomic analysis

Based on our screen results we chose mutants in 100 genes for proteomic profiling (Figure S2A; Table S3). The list includes 3 poorly-characterized genes that were not in the final hits but are hits in other data sets: *PSR23* and *PIIR2* are high confidence genes in Li et al.,^25^ and *PSR24* is a hit in 2 hit lists: low confidence in Li et al.^25^ and in Wakao et al.^21^

We grew starter cultures in TAP dark for about a week, then moved them to ~700 ml of TAP (initial concentration ~ 10^5^ per ml) in 1L bottles and continued growth in the dark. We bubbled air into the bottles and stirred them (using a magnetic stirrer) set to 200 RPM until they reached ~2x10^6^ cells ml^−1^. We pelleted ~5X10^7^ cells in 50 ml falcons, transferred the pellets to 1.5 ml tubes, pelleted them again, froze them on dry ice, and stored them at −80 °C.

For each proteomic 11-plex, we prepared 10 samples + a wild-type control. The wild-type control we used in most 11-plexes had been previously harvested in one experiment and frozen in aliquots to reduce the noise between the experiments.

We designed the experimental pipeline and our analysis to reduce the likelihood that artifacts would impact our conclusions:

1. We measured the overall protein abundance in each sample before we mixed them into the 11-plex to reduce the chance that one sample will dominate the 11-plex.
2. We focused on groups of proteins (like PSI proteins or Rubisco complex); the chance that an artifact will impact an entire complex is extremely low.
3. Each peptide is analyzed independently in the mass spectrometer, so proteins quantified from several peptides are much more reliable. The proteins we focused on, in most cases, are quantified from multiple peptides (e.g., the number of quantified peptides for PsaA is 8-9, PsaF is 4-8, PetA is 8-14, PetC is 3-5, PsaB is 12-20, PsaC is 9-15, AtpA is 14-18, and AtpB is 17-21) and thus, these proteins are more likely to be quantified accurately.
4. To reduce the chance that the specific set of mutants in an 11-plex will affect the results, the mutants were selected at random, and the replicate for each mutant was in an 11-plex containing a different set of other mutants.
5. If we observed a contradictory effect between the two repeats or one repeat showed a strong effect and the other didn’t show any, we assumed that one of them was an artifact and added an experimental repeat. In Figures 4B–4I, we count mutants as having a ‘‘proteomic phenotype” only if two repeats showed a similar phenotype.

### Sample processing and mass spectrometry

TMT-labeled (11-plex) peptides were prepared mostly as previously described.^91^ Frozen cell pellets were resuspended in 6 M guanidine hydrochloride (GdCl), 2% cetyltrimethylammonium bromide (CTAB), 50 mM HEPES, 1mM EDTA, and 5mM dithiothreitol (DTT) (pH 7.4). The resuspension lyses the algae to visual homogeneity. Mutant algae cultures grow to different densities and generate pellets of different mass. Diversity in pellet mass was normalized by diluting cells to that of the least dense culture by visual inspection. The final volume ranged from 200-1200 μL. 200 μL of each resuspension was removed to a new Eppendorf prechilled on ice. The lysed algae were sonicated at 20% power for 25 s. Proteins were denatured further at 60 °C for 20 min. After cooling, cysteines were alkylated by the addition of 20 mM N-ethylmaleimide for 30 min, followed by quenching with DTT (10 mM).

The protein solutions (200 μL) were charged with 800 μL MeOH, vortexed for 1 min, supplemented with 400 μl chloroform, vortexed for 1 min, followed by addition of 600 μl water and vortexing (1 min). The precipitated proteins were brought to the extraction interface by centrifugation (2 min, 20,800 x g), followed by removal of the upper layer. The protein interface was washed and pelleted from the chloroform phase by the addition of 600 μl MeOH, followed by vortexing (1 min) and centrifugation as described above. The wash solution was removed, and the pellet was washed with 1 ml MeOH. After the removal of MeOH, the pellets were resuspended in 50 μL of 6 M GdCl and 10 mM EPPS (3-[4-(2-hydroxyethyl)-1-piperazinyl]propane sulfonic acid) (pH 8.5). The resuspended pellets were frozen.

Pellets were thawed and their protein concentrations quantified using the BCA assay from Pierce with the BSA standard curve diluted in 10 mM EPPS pH 8.5 6M GdCl. 30 μg of each pellet was diluted to 15μL with 10mM EPPS pH 8.5 in 6M GdCl. The 15 μL of 2 μg/μL denatured protein solution was diluted with 75 μL 20 ng/μL LyseC in 10mM EPPS pH8.5, vortexed and allowed to digest overnight at room temperature. A second round of digestion followed with the addition of 270 μL of 20 ng/μL each LyseC and Trypsin in 10 mM EPPS pH 8.5, vortexing and overnight incubation at 37C. The solvent was removed under reduced pressure in a SpeedVac and resuspended in 30 μl of 200 mM EPPS (pH 8.0) to a concentration of 1 g/L. Ten microliters were removed from each resuspension and charged with 2μl of different TMT-isobaric mass tag N-hydroxysuccinimide (NHS) ester (20 g/liter). The acylation proceeded overnight at RT and was quenched at RT with 0.5 μL of 5% hydroxylamine for 20 min, followed by 1 μL of 5% phosphoric acid.

Peptides were enriched from the acidified TMT labeling reactions by solid-phase extraction using a Waters Oasis HLB Elution 96-well plate (3 mg/well). One well per multiplexed quantitative proteomics experiment was wetted with 400 μL MeOH and then hydrated with 200 μL water. The 11 labeling reactions are pooled and diluted into 400 μl and allowed to adsorb HLB resin under gravity flow. The adsorbed peptides were washed with 100 μL water, followed by centrifugation for 1 min at 180 rpm. The peptides were eluted with sequential additions of 100 μl of 35% acetonitrile (1% formic acid [FA]) and 100 μl of 70% acetonitrile (0.1% FA). Eluent solvent was removed under reduced pressure in a SpeedVac. The peptides were resuspended in 20 μL of 1% FA and subjected to quantitative multiplexed proteomics by nano-ultraperformance liquid chromatography-tandem mass spectrometry (nanoUPLCMS/MS).

Peptides were separated on a 75 μm inner diameter microcapillary column. The tip for the column was pulled inhouse and the column was packed with approximately 0.5 cm (5 μm, 100 Å, Michrom Bioresources) followed by 40 cm of Waters BEH resin (1.7 μm, 120 Å). Separation was achieved by applying a 3–22% Acetonitrile gradient in 0.125%, formic acid with 2% DMSO over 165 min at ~300 nL/min. Electrospray ionization was enabled by applying a voltage of 2.0 kV through an IDEX high-pressure fitting at the inlet of the microcapillary column.

TMT3 data collection was performed as previously described^91^ on a Fusion Lumos Tribrid Mass Spectrometer (Thermo). The instrument was operated in data-dependent mode (10 ions/scan) with an MS1 survey scan performed at a resolution setting of 120k (m/z 200) with a scan range of m/z 350 to 1,350, an RF (radio frequency) lens of 60%, automatic gain control (AGC) target of 1e^6, and a maximum injection time of 100 ms. Ions with charge states 2-6 were filtered by intensity with a threshold of 5e3. A dynamic exclusion window of +/−10ppm for 90s was used. MS2 quadrupole isolated ions (0.5 isolation window) were activated with CID at 35% collision energy and Q 0.25 and analyzed in the ion trap with an AGC target of 1.5e4 and 75ms maximum injection time. 10 data dependent MS3 synchronous precursor selections (2 isolation window) were selected from range 400-2000 m/z. The MS3 activation is HCD with 55% collision energy. The ions are analyzed in the orbitrap at 50,000 resolution with an AGC of 1.5e5 and an maximum injection time of 100 ms.

The proteomic (mass-spectrometry) data analysis is described in “mass spectrometry data analysis” in the “quantification and statistical analysis” section.

### Western blotting

Cultures were grown as for the proteomics experiment. 100 μL of cells (1-2 × 10^6^ cells mL^−1^) were lysed directly in 100 μL of 2x Laemli Sample Buffer (BioRad) + 5mM DTT, boiled at 95 °C for 10 min, and sonicated 3 s at 45% amplitude. Cell lysates were separated on a 10% SDS-polyacrylamide gel (BioRad), and transferred to a PVDF membrane using a semi-dry transfer system (BioRad). Membranes were blocked in 5% milk in PBS-T for 1 hour. The indicated primary antibody (PsbA - AS05 084A, Agrisera; PsbC - AS11 1787, Agrisera; PsaA - AS06 172, Agisera; ATPC - AS08 312, Agrisera; AtpB - AS05 085, Agrisera) was added and incubated with shaking overnight at 4°C, followed by three washes in 1xPBS-0.1% Tween. Secondary antibody was added for 1 hour at room temperature, followed by three additional washes in PBS-T. Blots were imaged using ECL reagent on an iBright imaging system. To control for total protein levels, we again washed the blots 3x 5 min in PBS-T and re-blotted overnight for α-tubulin (AS10 680, Agrisera).

### Chloroplast transcriptome profiling (Chloroplast RNAseq)

The RNA seq experiments were split into two experiments; each experiment had its own wild type. In each experiment, we had 2-3 replicates for each mutant strain and 2-4 replicates for the wild type.

The strains were grown in the same conditions as for the proteomic analysis. When the cultures reached ~ 2x10^6^ cells/ml, we pelleted 13 ml of culture in 15 ml round Falcon tubes. We then used TRIzol extraction (following the manufacturer’s protocol) to obtain the total RNA. The RNA was sent to the Princeton Genomics Core Facility for RNAseq and Next Generation Sequencing. The chloroplast mRNA does not have polyA, so the facility used the Qiagen FastSelect – rRNA Plant Kit for rRNA depletion. The facility then used the PrepX^™^ RNA-Seq for Illumina Library kit to generate the library for RNAseq.

mRNA analysis: First, non-coding RNA sequence was filtered out: each dataset was aligned (using the bowtie2 –fast command) against the dataset of non-coding RNAs,^93^ and only unaligned reads were included in the rest of the analysis. Next the reads were aligned against a reference file containing the updated chloroplast and mitochondrial genomes,^93^ a set of Chlamydomonas rRNA sequences (downloaded from https://www.arb-silva.de/), and Chlamydomonas nuclear coding sequences (v5.5 from Phytozome, file Creinhardtii_281_v5.5.cds_primaryTranscriptOnly.fa), using the bowtie2 –fast option. For each sample, the number of reads in each chloroplast gene was calculated in python, with each side of each read considered separately, and with gene positions based on the chloroplast gff3 file from Gallaher et al.^93^

The reads were used to estimate the mRNA levels of the different chloroplast-expressed photosynthetic genes. The reads were normalized by the total chloroplast gene reads.

Our RNA seq reads were paired-end, allowing us to estimate splicing efficiency by analyzing where each side maps on the genome: when paired reads mapped to adjacent exons, the intron between them was considered spliced out. If the read in one end was in exon 1 and the read in the second end was in exon 3, this read was considered to be from a fully-mature mRNA. The overall coverage was much higher in our second experiment, so we normalized the 1^st^ experiment using the wild-type ratio between the experiments, allowing us to present them together.

### Nuclear RNAseq

The mRNA of *pmr1* (2 independent experiments) and wild type (2 independent experiments) was also used for polyA-based RNAseq. The library preparation and Next Generation Sequencing were done at the Princeton Genomics Core Facility.

The paired-end reads were aligned against the primary transcriptome (v5.5, from Phytozome) using the bowtie2 –fast command, and the number of reads aligning to each transcript were counted in python for each sample.

We normalized the number of reads to 50M, then we averaged (using the geometric mean) the 2 experimental repeats of *pmr1* and the 2 experimental repeats of wild type, and then calculated the relative reads by log_2_(pmr*1*/ wild type).

### Measurement of NADP^+^ and NAD^+^ in wild type and *pmr1* mutant (in-vivo)

We used liquid-chromatography mass spectrometry to measure the cellular levels of NADP^+^ and NAD^+^ in wild type and *pmr1* mutant. The protocol was adapted from Yuan et al.^94^ In short, we grew starter cultures at TAP dark for about a week, then inoculated experimental cultures in ~700ml of TAP in 1L bottles at an initial concentration ~ 10^5^ per ml. We grew the experimental cultures in the dark stirred using a magnetic stirrer at 200 RPM and bubbled with air until they reached ~2x10^6^ cells ml^−1^. We harvested ~ 10^7^ cells using vacuum filter, and immediately dunked the filter’s membrane into 1.5 ml of 40:40:20 (v/v/v) methanol:acetonitrile:H_2_O solution with 0.5% formic acid to extract the metabolites. All reagents were precooled to −20 °C and the protocol was performed on ice. After neutralizing by NH_4_HCO_3_ (132 μL) and pelleting, we took 100 μl supernatant for LC-MS.

The LC-MS method was modified from Yang et al.^95^ Water-soluble metabolite measurements were obtained by running samples on the Orbitrap Exploris 480 mass spectrometer (Thermo Scientific) coupled with hydrophilic interaction chromatography (HILIC). An XBridge BEH Amide column (150mm X 2.1 mm, 2.5 uM particle size, Waters, Milford, MA) was used. The gradient was solvent A (95%:5% H_2_O:acetonitrile with 20 mM ammonium acetate, 20 mM ammonium hydroxide, pH 9.4) and solvent B (100% acetonitrile) 0min,90% B; 2min,90% B; 3min,75% B; 7min,75% B; 8min,70% B; 9min, 70%B; 10 min, 50% B; 12 min, 50% B; 13 min, 25% B; 14 min, 25% B; 16 min, 0.5% B, 20.5 min, 0.5% B; 21 min, 90% B; 25 min, 90% B. The flow rate was 150 μL/min with an injection volume of 5 μL and a column temperature of 25 °C. The MS scans were in polarity switching mode to acquire data from both positive and negative ions across a mass range of 70–1000 m/z, with a resolution of 120,000. Data were analyzed using the EI-MAVEN software (v 0.12.0, Elucidata).

We included a total of 3 replicates from each strain from 2 independent experiments.

### Protein purification

The *pmr1* rescued cells expressing PMR1-Venus-3xFLAG and the control cells expressing Venus-3×FLAG were pre-cultured in 50 mL TAP medium with 5 μmg mL^−1^ until the cell density reached ~2–4 ×10^6^ cells mL^−1^. Then, the culture was diluted into 1,000 mL TAP liquid medium to a concentration of ~2 ×10^4^ cells mL^−1^. Cells were grown with air bubbling and constant stirring at 210 RPM under 150 μmol photons m^−2^ s^−1^ light until the cell density reached ~2-4 ×10^6^ cells mL^−1^. Cells were collected by centrifugation at 3,000 g for 4 min in an Avanti J-26X centrifuge with an 8.1000 rotor (Beckman) at 4 °C. The pellets were washed in 35 mL ice-cold washing buffer (25 mM HEPES, 25 mM KOAc, 1 mM Mg(OAc)_2_, 0.5 mM CaCl_2_, 100 mM Sorbitol, 1mM NaF, 0.3 mM Na_3_VO_4_, and complete EDTA-free protease inhibitor (1 tablet/500 mL)) and then resuspended in a 1:1 (v/w) ratio of ice-cold 2×IP buffer (50 mM HEPES, 50 mM KOAc, 2 mM Mg(OAc)_2_, 1 mM Cacl_2_, 200 mM Sorbitol, 1mM NaF, 0.3 mM Na_3_VO_4_, and cOmplete EDTA-free protease inhibitor (1 tablet/50 mL). 3 mL cell slurry was immediately added to liquid nitrogen to form small popcorn pellets which were stored at −80 °C until needed. Cells were lysed by cryogenic grinding using a Cryomill (Retsch) at frequency of 25 oscillations per second for 20 min. The ground powder was defrosted on ice for 45 min and dounced 25 times on ice with a Kontes Duall #22 homogenizer (Kimble). Proteins were solubilized by incrementally adding an equal volume of ice-cold 1×IP buffer plus 2 % digitonin (RPI) followed by an incubation of 45 min with nutation at 4 °C. The cell debris were removed by spinning at 12,700 x g for 30 min at 4°C. The supernatant was then mixed with 50 μL anti 3×FLAG magnetic beads (Sigma) which had been previously washed sequentially with 1×IP buffer 3 times and 1×IP buffer plus 0.1 % digitonin 2 times. The mixture was incubated with nutation at 4 °C for 1.5 hr, followed by the removal of supernatant. The beads were washed 4 times with 1×IP buffer plus 0.1 % digitonin followed by a 30 min competitive elution with 45 μL of storage buffer (20 mM HEPES, pH7.4, 350 mM KCl, 1 mM EDTA, 10% (vol/vol) glycerol, and 5 mM DTT) and 2 μg/μL 3×FLAG peptide (Sigma-Aldrich). Protein purity was assessed by SDS-PAGE followed by Coomassie blue staining.

### Measurement of NADP(H) dephosphorylation activity of PMR1 in vitro

The NADP(H) dephosphorylation reaction was carried out at 22 °C using 1 mM NADP(H) (Roche) and 0.5 μM Nocturnin, PMR1-Venus-3xFLAG, or Venus-3xFLAG. Reactions contained 20 mM Tris⋅HCl (pH 8.0), 70 mM NaCl, and 2 mM MgCl2. At the indicated time points, the reaction was quenched using 4 volumes of cold methanol, and then further diluted 100-fold with methanol before LC-MS analysis. The LC-MS conditions were the same as in the in-vivo experiment, except that we used an Exploris 240 mass spectrometer, and the mass range of 600–800 m/z was scanned. In our conditions the dominant form of NADP(H) was NADP^+^ (~98%), so we followed this form in the experiment.

### Indirect Immunofluorescence Assay

Indirect immunofluorescence was performed as described previously.^96^ First, cells were harvested by centrifugation and rinsed with PBS buffer twice. Next, 100 μL of cells were spotted onto Poly-L-lysine-coated glass slides (Sigma-Aldrich). Cell fixation was done by 4 % (w/v) formaldehyde (Sigma-Aldrich) in PBS for 20 min and then incubated with 100 % ice-cold methanol for 20 min to remove chlorophyll. Purified antibodies (Yenzyme) against PMR1 were used at a dilution of 1:50. The purified antibodies were generated using the following peptide: C-Ahx-EGRSFQDDSTGREQSQGY-amide. After washing the slides six times, each with 50 mL PBS-T (with 0.1% Tween 20 (v/v)) in a Coplin jar, Alexa Fluor 488 goat anti-rabbit IgG (H+L) Cross-Adsorbed Secondary Antibody (Invitrogen) was used at a dilution of 1:500. The slides were washed six times, each with 50 mL PBS-T. Fluorescence and bright-field images were acquired using a confocal microscope (Leica, SP5).

## QUANTIFICATION AND STATISTICAL ANALYSIS

The data quantification and overall statistical analysis were done using MATLAB. The sequencing data were analyzed by Cutadapt, Bowtie 2, and python. The confocal images were analyzed by Fiji. The structural data were analyzed and displayed using PyMOL.

The number of experimental repeats (n) is provided in the legends of the corresponding Figures. The error bars represent standard deviation (SD) and are described in the legends. The definition of center (mean or median) is described in the legends.

### Barcode quantification, normalization, and growth score calculation

We trimmed the initial reads using cutadapt version 1.18. Sequences were trimmed using the command “cutadapt -a <seq> -e 0 -q 33 -m 21 -M 23”, where <seq> is GGCAAG for 5′ data and TAGCGC for 3′ data. Next, The barcode read counts for each dataset were calculated in python, filtered to only include barcodes present in the original library,^25^ and normalized to a total of 1 million.

We calculated the “normalized light growth after backcrossing” metric as follows:

1. We used the correlation between the different experimental repeats of each condition to check for swapped samples. Based on these results, we corrected 2 swapped sample pairs: (1) TAP dark sample 3 from Exp3A (MK 1-12 rep2), with TP light sample 1 from Exp2B (MK13-26 rep1); (2) TAP dark sample 1 of NP biological replicate 1, with TAP dark sample 3 of NP biological replicate 2.
2. We averaged the read count of each barcode across the different replicate samples for each condition, using median if we had three replicates or geometric mean if we had only two.
3. To reduce the noise, we removed samples with very low read counts in the TAP condition (<7 in the first experiment and <10 in the rest).
4. We calculated the relative growth as log_2_ (averaged TP light reads / averaged TAP dark reads). In the first experiment, we had two different conditions; one was grown in hygromycin and paromomycin, and the other only in hygromycin; we analyzed them separately.
5. We normalized the NP experiment results – the overall distribution of relative growth rates in the NP experiment was shifted because most of the strains in this competition have a photosynthetic defect, so we scaled the results from this experiment by 0.6 to get a similar distribution to the other experiments.
6. For the final “growth score,” we used the median of the five experiments with the strongest photosynthetic growth defects (for all but 122 genes, it is the same as using all the data). We used the five experiments with the photosynthetic growth defects because there are slightly different conditions between experiments, which can affect the phenotype. Furthermore, in some repeats, we were unable to see an effect because we did not manage to remove all the diploid cells. Lastly, the possibility that the mutants will have a phenotype “by chance” in more than five different experiments is very low, so even slightly lower effects for genes with many experimental repeats can be tolerated. The growth score and the light/dark ratio of backcrossing experiments for all the strains are shown in Table S1.
  We used the “growth score” to set the 0.34 threshold to identify hits and to calculate the FDR (see below, and Figure S1). To reduce noise, we counted as hits only the strains that had reads above the threshold in at least two experiments.
7. FDR calculation (see also Figure S1) – to calculate the False Discovery Rate (FDR) we first estimated how many of the 1,616 mutated genes in our starting set are required for photosynthesis. We sampled 350 genes at random from the 1,616 and searched the literature for genes among them that are required for photosynthesis. Approximately 6.25% of the genes were known to be required for photosynthesis. Considering previous estimates indicating that approximately half of the genes required for photosynthesis remain to be discovered,^25^ we estimate that an additional 6.25% of the genes in the initial set are also required for photosynthesis; thus, we estimate that 12.5% of the initial genes are required for photosynthesis, and the remaining 1,414 (87.5% of the initial 1,620 genes) in our starting set are not required for photosynthesis. Next we defined a set of genes that we called “Genes whose disruption likely did Not Result in a Photosynthesis Defect” (GNRPD). We assigned genes from our set of 1,616 to GNRPD if they were represented by more than 20 insertions, where at most two mutants showed a photosynthetic defect in the Li et al. experiment. We selected the threshold of 0.34 as a compromise between low false-discovery rates and a relatively large number of hits. A phenotype threshold of 0.34 resulted in 136 hit genes identified, which included 2/204 (~1%) of the GNRPDs. We assume that the same percentage (~1%) of the 1,414 estimated genes in our starting set that are not required for photosynthesis in the original mutant set, will go into the hits, resulting in a calculated FDR < 0.11 when using a threshold of 0.34. With a threshold of 0.49, the same calculation yields 227 hit genes with an FDR < 0.3.

As a sanity check for the FDR calculation, we also calculated the hit p-value based on linkage distance (Figure S1).

### Mass spectrometry data analysis

Mass spectrometry raw data were analyzed using GFY software licensed from Harvard^92^ to quantified proteins relative abundance.

We normalized each protein’s abundance in each sample by that protein’s abundance in the corresponding wild-type/control sample, then normalized the protein’s abundance in the sample by the sample’s median to account for systematic difference likely coming from technical difference in the amounts of proteins entered into the TMT labeling.

To decrease the noise, we used 11-plex-median-based normalization (Figure S4). We divided the abundance of each protein in a given sample by the median abundance of this protein in its 11-plex. This normalization sets the median of each 11-plex to 1 on a linear scale (0 on a log scale). This normalization process intends to correct two kinds of artifacts: 1) when one protein is over(/under)-represented in all samples of one specific 11-plex (as in the case of S4A); and 2) to set the overall median relative abundance of this protein across all 11-plexes to 1 on a linear scale (0 on a log scale), to control for systematic effects. Systematic effects, such as the underrepresentation of ribosomal proteins in the data before the 11-plex-median-based normalization visible in Figure S4E, are likely due the reference wild-type control strain that was included in the 11-plexes and used to calculate the proteins relative abundance. This normalization improves the overall quality of the data, as seen in Figure S4.

We are aware that if most of the mutants in a group have similar proteomic effects, the median normalization could lead to the over- or underestimation of the abundance of that protein. This effect is rare because the mutants were selected at random, and a change in the median value would require five of the mutants to have a similar effect on the proteome. Furthermore, we randomized the mutants present in the 11-plexes of the two repeats. If the two repeats disagreed, we assumed there was an experimental problem and performed an additional repeat. Thus, it is unlikely that our normalization strategy would produce significant artifacts in the proteomics data shown in the figures.

## Supplementary Material

MMC6

MMC1

MMC4

MMC5

MMC2

MMC3

1

## Figures and Tables

**Table 1.** Protein localizations and suggested functions of the rescued genes^83^

Systematic ID	Name	Figures	Localization	Suggested function
*Cre17.g728850*	RAA15	Figures 2C, 4, and 5	predicted mitochondrion	our proteomics and transcriptomics data suggest that this protein participates in splicing of the 2^nd^ intron of *psaA* mRNA (see main text)
*Cre13.g566400*	RAA17	Figures 2D, 4, and 5	predicted mitochondrion	our proteomics and transcriptomics data suggest that RAA17 stabilizes the 3^rd^ exon of *psaA* (see main text)
*Cre10.g448950*	PMR1	Figures 6 and 4	chloroplast, cytosol, nucleus	photosynthesis master regulator 1—our proteomics and metabolomics data suggest that PMR1, a nocturnin homolog (Figure S8), participates in retrograde regulation and affects mRNA levels of ROGEs (see main text)
*Cre12.g560550*	MTF1	Figures 6 and 4	chloroplast	methionyl-tRNA formyltransferase 1—our data indicate that MTF1 is the chloroplast methionyl-tRNA formyltransferase and suggest that it participates in the regulation of the chloroplast-expressed genes (see results and discussion)
*Cre12.g485850*	CPLD64	Figures 2E and 4	predicted chloroplast	in our data, the *cpld64* mutant showed depletion of the cytochrome *b*_6_*f* complex; CPLD64 has a predicted transmembrane motif (InterPro: IPR009688); these observations suggest that CPLD64 participates in the biogenesis or stability of the cytochrome *b*_6_*f* complex in the thylakoid membrane
*Cre01.g014000*	PIR1	Figures 2F, 2P, 4, and 5	chloroplast	photosystem I required 1—in our proteomics data, the *pir1* mutant showed depletion of PSI; our RNA-seq data suggest that PIR1 does not participate in the *psaA* mRNA maturation process; it may participate in PsaA or PsaB translation
*Cre06.g279500*	CPL6	Figures 2J, 2Q, and 4	chloroplast	CPL6 contains a DnaJ heat shock protein domain; we observed that the *cpl6* mutant did not exhibit depletion of any photosynthetic complex, suggesting that its chaperone activity is not needed for complex formation in the dark; *cpl6* cannot grow under high-light conditions even when supplied with a carbon source (acetate), suggesting that CPL6 may contribute to repairing light damage to the photosynthetic machinery
*Cre02.g073850*	CGL54	Figures 2H, 2R, and 4	pyrenoid periphery	CGL54 is in the same protein superfamily as cyanobacterial Psb27 (Figure S9), which is involved in PSII biogenesis^83^; however, a different gene, PSB27, shows higher homology to Psb27 (Figure S9) and the *cgl54* mutant did not lead to the depletion of PSII, suggesting that CGL54 has a different function; CGL54 localized to the pyrenoid periphery, similarly to the PSI-interacting protein PSBP4,^30^ suggesting that CGL54 may interact with PSI
*Cre10.g433400*	PSR1	Figures 2l and 4	predicted other	photosynthesis required 1 is a homolog of the mitochondrial pyruvate carrier (InterPro: IPR005336); the photosynthetic defect of the *psr1* mutant was alleviated under high CO_2_,^24^ suggesting that PSR1 participates in the CO_2_-concentrating mechanism (CCM)
*Cre10.g466500*	CPL12	Figures 2J, 2S, and 4	chloroplast	CPL12 belongs to the glyoxalase I family (KEGG: K08234); it may participate in the detoxification process of methylglyoxal, a byproduct of photosynthesis^84^
*Cre01.g040050*	TPK1	Figure 2K	predicted other	TPK1 is the *Chlamydomonas* homolog of thiamine pyrophosphokinase (KEGG: K00949); TPK1’s photosynthetic effect is likely due to the participation of TPK1 in the chloroplast pentose phosphate pathway
*Cre01.g022681*	PSR5	Figure 2L	predicted other	PSR5 is a small protein, and its expression is light inhibited^41^

**Table:** KEY RESOURCES TABLE

REAGENT or RESOURCE	SOURCE	IDENTIFIER
Antibodies		
Rabbit anti- PsbA	Agrisera	Cat# AS05 084A; RRID: AB_2172617
Rabbit anti- PsbC	Agrisera	Cat# AS11 1787
Rabbit anti- PsaA	Agrisera	Cat# AS06 172; RRID: AB_2237771
Rabbit anti- ATPC	Agrisera	Cat# AS08 312; RRID: AB_2290280
Rabbit anti- AtpB	Agrisera	Cat# AS05 085; RRID: AB_2258955
Rabbit anti- α tubulin	Agrisera	Cat# AS10 680; RRID: AB_10748620
Goat anti-Rabbit IgG(H+L) Highly Cross-Adsorbed, Alexa Fluor 488	Invitrogen	Cat# A11034
Rabbit polyclonal anti-PMR1	This paper	N/A
Chemicals, Peptides, and Recombinant proteins		
UltraPure Low-Melting Point Agarose	Invitrogen	Cat# 16500100
TRIzol^™^ Reagent	Invitrogen	Cat# 15596026
cOmplete, EDTA-free Protease inhibitor	Roche	Cat# 5056489001
Anti-FLAG M2 Magnetic Beads	Sigma-Aldrich	Cat# M8823
3×FLAG peptide	Sigma-Aldrich	Cat# F4799
4×Laemmli sample buffer	Bio-Rad	Cat# 1610747
Guanidine hydrochloride	Sigma-Aldrich	Cat# 369080-1KG
Cetyltrimethylammonium bromide	Sigma-Aldrich	Cat# 57-09-0
HEPES	Sigma-Aldrich	Cat# H3375-25G
NEM	Sigma-Aldrich	Cat# 128-53-0
DTT	Sigma-Aldrich	Cat# 3483-12-3
EPPS	Sigma-Aldrich	Cat# 000010
Methanol	Fisher scientific	Cat# A456-4
Acetonitrile	Fisher scientific	Cat# A955-4
Chloroform	VWR	Cat# BDH83626.400
H_2_O	VWR	Cat# 87003-652
Formaldehyde solution 4 %, pH6.9	Sigma-Aldrich	N/A
Formic acid	Sigma-Aldrich	Cat# F0507
NH4HCO3	Sigma-Aldrich	Cat# 09830
MAX efficiency transformation Reagent for Algae	Invitrogen	Cat# A24229
Critical Commercial Assays		
Phusion High-Fidelity DNA polymerase	New England BioLabs	Cat# M0530L
MinElute Gel Extraction Kit	QIAGEN	Cat# 28606
Gibson Assembly Master Mix	NEB	Cat# E2611L
MT10plex Isobaric Label Reagent Set plus TMT11-131C Label Reagent	Thermo	Cat# A34808
QIAprep Spin Miniprep Kit	QIAGEN	Cat# 27106
Experimental Models: Organisms/Strains		
*C.reinhardtii*: wild-type CC-4453	Chlamydomonas Resource Center	CC-4533 cw15
*C.reinhardtii*: wild-type CC-1690	Chlamydomonas Resource Center	CC-1690
CLiP library mutants in Table S1	Li et al.^25^; Chlamydomonas Resource Center	https://www.chlamycollection.org/
*E.coli* Stellar Competent Cells	Takara	Cat# 636763
Chlamydomonas rescued strains listed in “mutant gene rescue protocol” section	This paper, Chlamydomonas Resource	https://www.chlamycollection.org/
Oligonucleotides and Recombinant DNA		
pLM005	Mackinder et al.^85^	GenBank: KX077945.1
pRAM118	Itakura et al.^86^	GenBank: MK357711
Plasmid constructs generated and listed in “mutant gene rescue protocol” section	This paper, Chlamydomonas Resource Center	https://www.chlamycollection.org/
Software and Algorithms		
MATLAB	MathWorks	N/A
Python	N/A	N/A
PyMOL	pymol.org	https://pymol.org/2/
Bowtie 2	Bowtie	http://bowtie-bio.sourceforge.net/bowtie2/manual.shtml
Cutadapt 1.18.	cutadapt	https://cutadapt.readthedocs.io/en/v1.18/
Fiji	Schindelin et al.^87^	https://imagej.net/software/fiji/downloads
Other		
Electroporation Cuvette, 2mm gap	Bulldog Bio.	Cat# 12358-346
Ibidi USA μ–Slide 8 well, Glass bottom	Ibidi	Cat# NC0704855
Poly-L-lysine coated glass slides	Sigma-Aldrich	Cat# P0425
Kontes Duall #22 homogenizer	Kimble	Cat# KT885450-0022
Cryomill	Retsch	Part NO. 20.749.0001
10% Mini-PROTEAN^®^ TGX^™^ Precast Protein Gels	BIO-RAD	Cat# 4561036
Lumigrow Lumibar lights	Lumigrow Lumibar	Cat# 8100-5502
Vacuum filter flask, with a fritted glass support base.	Wilmad Labglass	Cat# BP-1752-001
Nylon membrane filters (0.5μm pore size)	GVS Magna^™^	Cat# 1213776
Oasis HLB 96-well μElution Plate, 2 mg Sorbent per Well, 30 μm, 1/pk	Waters	Cat# 186001828BA
Electroporator	NEPA GENE	NEPA21 type II
SP5 Confocal Microscope	Leica	TCS SP5
Singer Rotor HAD	Singer Instruments	Cat# ROT-001
PhenoBooth imager	Singer Instruments	N/A
Nikon Confocal laser scanning Microscope	Nikon	A1Rsi
Typhoon FLA9500 fluorescence scanner	GE Healthcare	N/A
iBright imaging system	invitrogen	iBright 1500

## Highlights

- Identification of 70 poorly characterized photosynthesis genes in *Chlamydomonas*
- Proteomic analysis of mutants allows assignment of function to poorly characterized genes
- Characterization of 5 photosystem I mRNA maturation factors validates this resource
- MTF1 and PMR1 identified as master regulators of photosynthesis
