# Supplementary material for: Systematic identification and characterization of genes in the regulation and biogenesis of photosynthetic machinery
Source: Cell. Author manuscript; Available in PMC 2024 Jan 2. (PMC10760936; doi:10.1016/j.cell.2023.11.007)
Supplement: 1 [file NIHMS1945839-supplement-1.pdf]

Cell
Resource
CellPress
OPEN ACCESS

Supplemental figures

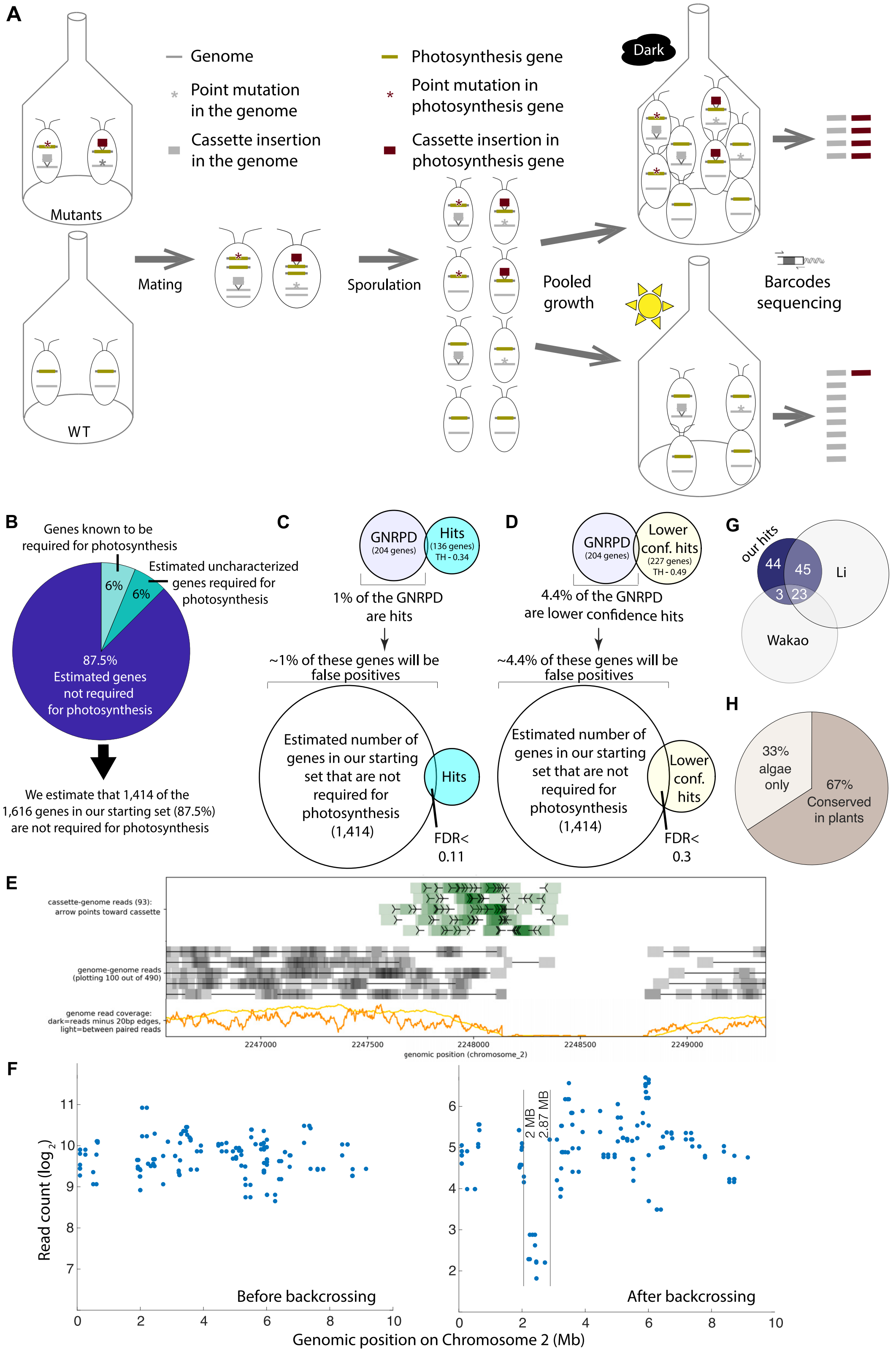

(legend on next page)

Figure S1. Pooled backcrossing and FDR calculation, related to Figure 1

(A) The mutants carried barcoded cassette insertions and additional mutations. To determine which barcoded cassette insertions are linked to the observed photosynthetic phenotype, we mated the mutants, which were paromomycin-resistant and mt−, with a hygromycin-resistant mt+ strain. The resulting progeny included mixed genotypes where the insertions and the second-site mutations segregated randomly. We grew the progeny under a dark control condition, where all viable strains grew, and photoautotrophically ($\sim$750 μE/m^2/s), where mutants in genes required for photosynthesis were depleted. By sequencing the pools of barcodes associated with insertions, we could identify barcodes that were depleted under the photoautotrophic condition and thus were genetically linked to genes required for photosynthesis.

(B) Calculation of the "estimated number of genes in our starting set that are not required for photosynthesis." Our dataset included 1,616 genes with mapping confidence level[25] <4. We sampled 350 genes at random from the 1,616 and screened the literature for genes among them that are required for photosynthesis. 6.25% of the genes were known to be required for photosynthesis. Considering previous estimates indicating that approximately half of the genes required for photosynthesis remain to be discovered,[25] we estimate that an additional 6.25% of the genes in the initial set are also required for photosynthesis; thus, we estimate that 87.5% of the genes in our starting set are not required for photosynthesis. Given these numbers, the estimated number of genes in our starting set that are not required for photosynthesis is 1,414 (87.5% of the initial 1,616 genes).

(C) The false discovery rate (FDR) calculation is based on a set of specific genes that we called genes whose disruption likely did not result in a photosynthesis defect (GNRPD). Genes from our set of 1,616 genes were assigned to GNRPD if they were represented by more than 20 insertions in Li et al. experiment and at most two mutants showed a photosynthetic defect. $\sim$1% of the GNRPDs were among the 136 hit genes identified with a phenotype threshold of 0.34. We assume that the same ratio ($\sim$1%) of the estimated number of genes in our starting set that are not required for photosynthesis (see B) in the original mutant set will go into the hits, yielding an estimated FDR < 0.11. In the manuscript, we focused on this threshold due to its low FDR and due to the shape of the distributions (Figure 1E): the GNRPD distribution goes down to almost zero below this threshold.

(D) The same calculation as (C) was repeated for lower-confidence hits (phenotype threshold of 0.49) and indicated that these lower-confidence hits have FDR < 0.3. Despite the higher false discovery rate, these hits still include many genes genuinely required for photosynthesis.

(E) We used whole-genome paired-end Illumina sequencing to identify the insertion locus of the hygromycin resistance cassette in the wild-type strain. We identified the insertion in chromosome 2 around position 2.25 Mb. Cassette-genome reads are chimeric paired reads where one read maps to the genome and the other maps to the cassette. Genome-genome reads are paired-end reads where both reads mapped to adjacent regions in the genome; such reads are depleted in the proximity of an insertion. The insertion appears complex, and the data are insufficient to fully map the insertion site with confidence, but a model consistent with the observed data is that there is a genomic inversion from $\sim$2248150 to $\sim$2248450, followed by the cassette insertion, followed by poorly mappable sequence, and then genomic sequence resuming at $\sim$2248800 (with a genomic deletion from $\sim$2248450 to $\sim$2248800).

(F) To estimate the linkage distance, defined as the minimum distance between two markers needed for independent segregation in our backcrossing, we evaluated barcode read counts from backcrossed pools grown in the dark on hygromycin, where all barcodes should be present except for the ones that were too close to the hygromycin cassette for recombination to occur. We measured the linkage distance as the distance from our HygroR marker to the edge of the region of depleted insertions. To reduce noise levels, we used only insertions with a mapping confidence level of 95% and smoothed the data by using a running median. This linkage distance was 0.25 Mb on one side of the mapped hygromycin cassette insertion and 0.6 Mb on the other side of the insertion. Note that the FDR metric we use is more conservative than a p value based only on linkage size. Even if we assume an upper-limit linkage region size of 2 Mb (1 Mb from each side), the chance for a random insertion to be inside the linkage region is <0.017 (2/120 Mb—*Chlamydomonas* genome size). Even if we assumed a worst-case scenario where each strain has four additional unknown mutations, the chance that all of them will be in the rest of the genome is >0.93 $(118/120)^4$, so the chance that at least one of them will be in the linkage region is <0.07, and thus, a p value based only on linkage size would be $p < 0.07$. We are thus more conservative by stating that our FDR is <0.11.

(G) 26 of our 115 hits (23%) were also hits in Wakao et al.,[21] and 68 of the 115 (59%) were also hits in Li et al.[25]

(H) More than 65% of our hits are conserved in land plants.

CellPress
OPEN ACCESS

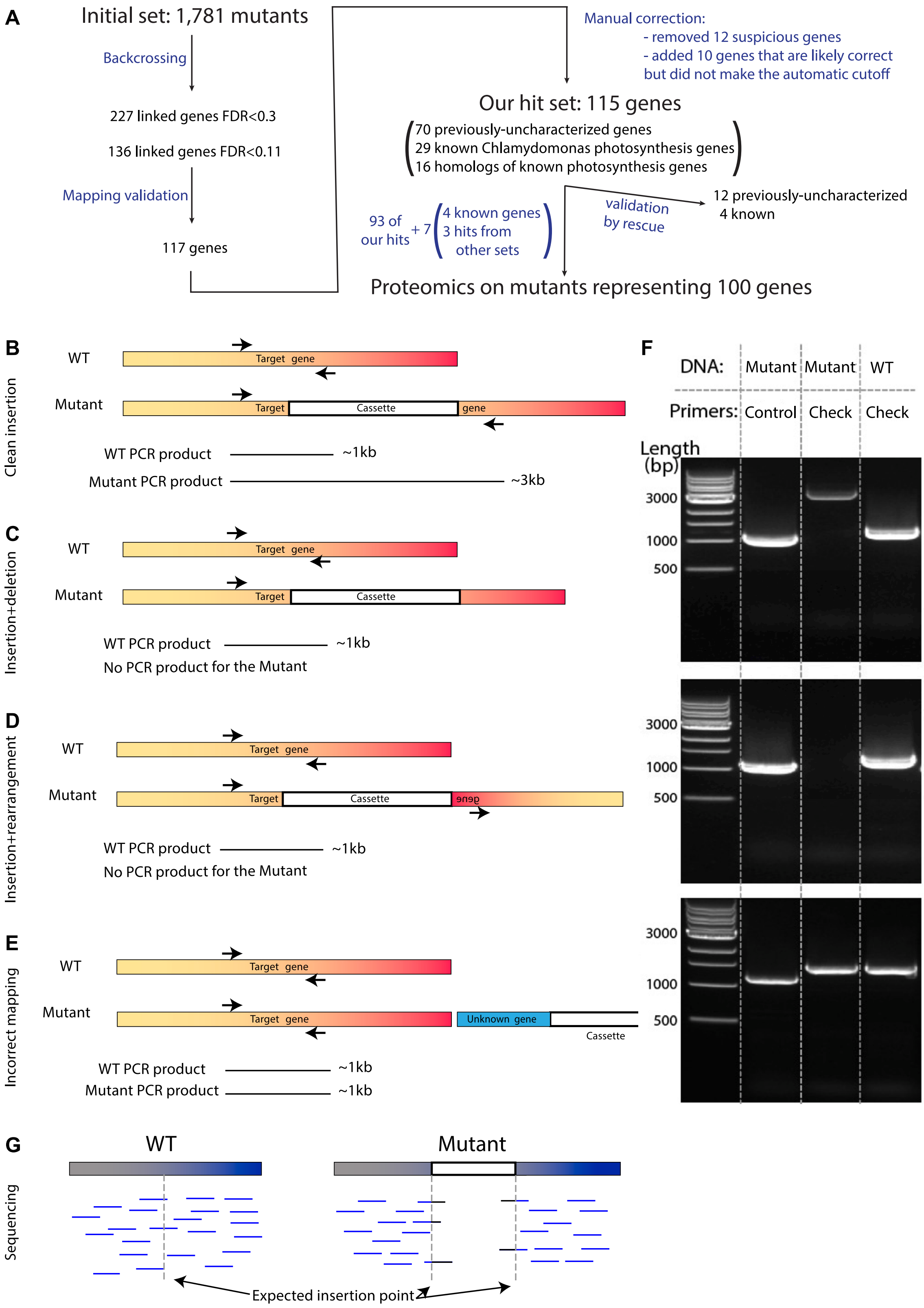

(legend on next page)

Figure S2. Supplemental data for the genetic screen, related to Figure 1

(A) Mutant and gene numbers at different stages of this project. For a detailed description of the process, please see the STAR Methods. Mutant and gene IDs are provided in Table S1.

(B–F) Mapping validation by colony PCR. Displayed are four scenarios of cassette insertion and the expected PCR product (B–E), and PCRs examples (F). (B) Clean insertion—the cassette integrates into the genome cleanly; in this situation, the PCR product of the mutant will be approximately 2 kb longer than the WT product. (C) Insertion with significant deletion—in this case, the deletion associated with the insertion removed one of the genomic PCR primer-binding sites; therefore, we will get the PCR product for WT but not from the mutants. (D) Insertion with rearrangement—in this case, the primer sequence is there but in the incorrect orientation, so again we will get a PCR product for WT but not for the mutant. (E) When the insertion is not in our expected gene, we will get the same length of PCR product from the WT and the mutants. (Note that we can get this pattern also if the insertion is associated with a deletion of a similar size.) (F) Example of colony PCR results. The control lane is mutant DNA amplified using control primers to verify the mutant DNA quality. In the upper example, the mutant is ~2 kb longer than the WT, as expected from a clean insertion (B). In the middle example, we have a band for the WT but not for the mutants. Such a result was interpreted as validating an insertion site if it was reproduced at least twice, and is expected for scenarios (C) and (D). The lower example was interpreted as a failure to validate the mapping and is expected for (E). When we failed to get a product with WT, we used different primers or whole-genome sequencing to map the insertion site.

(G) Mapping validation by sequencing. Mutant genomes were sequenced using Illumina paired-end 150 nt reads. We considered an insertion site validated when we found in the expected area chimeric paired-end reads (where one read mapped to the genome and the other to the cassette) and a “hole” in the genome coverage. For more details, see STAR Methods.

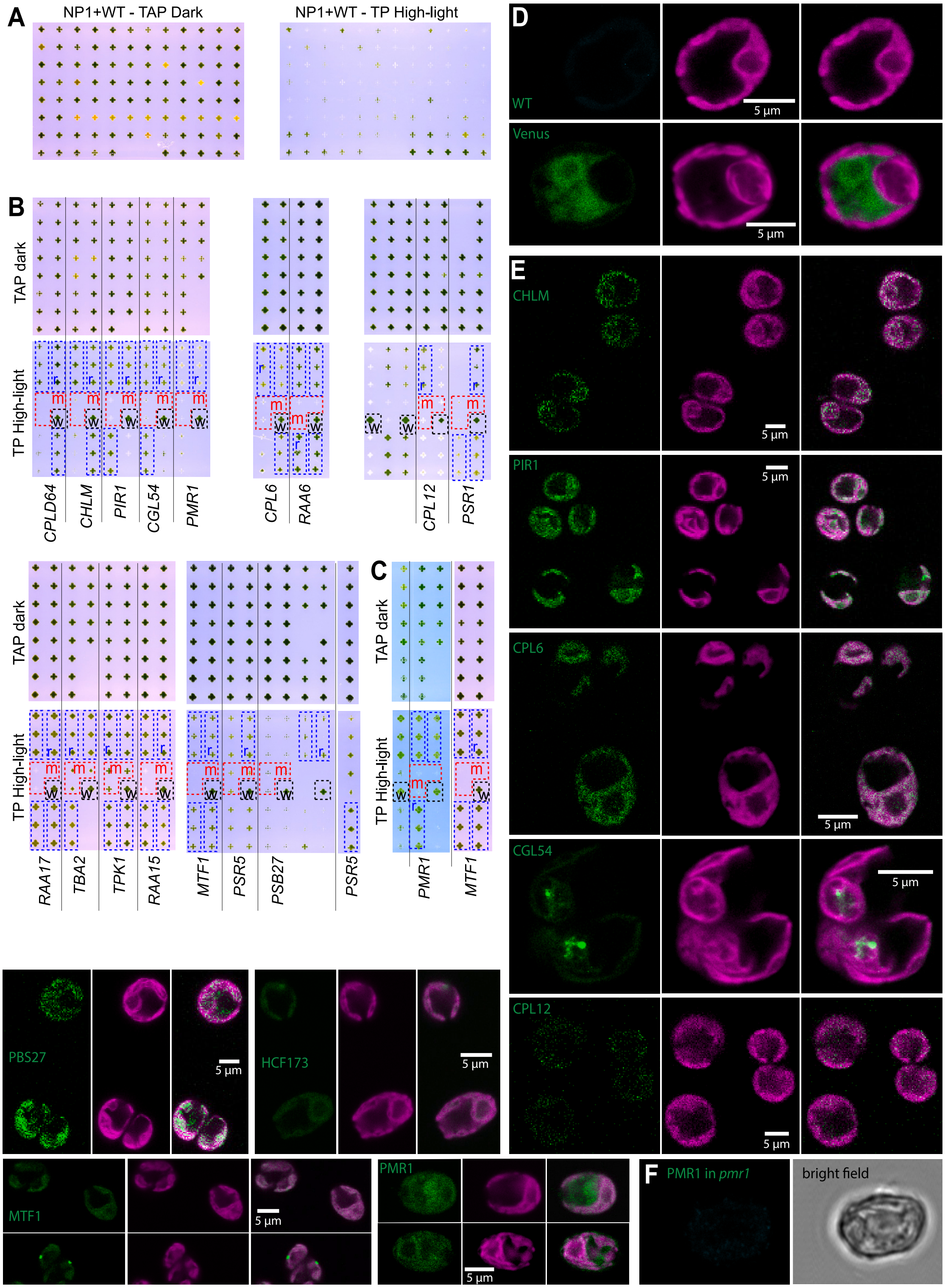

Figure S3. Additional images, related to Figures 1, 2, and 6

(A) Images for Figure 1B before background removal.

(B) Unprocessed plate images for Figure 2. In each high-light plate, the three copies of the original mutants are outlined in dashed red and every triplicate of the rescued strains is outlined in dashed black. To reduce the effect of location on the plate, we put one WT next to each mutant trio. The “r” indicates the rescued strain used in the main figure. Similarly, “m” indicates the mutants and “w” the WT used in the main figure. There are differences in the rescue efficiency between

(legend continued on next page)

the different rescued strains, even in the same mutant. Many parameters could contribute to those differences, including insertion site and expression level. The light level for the photosynthetic conditions was approximately 1,100 $\mu E/m^2/s$.

(C) The unprocessed plate images for Figure 6. The light levels for the photosynthetic conditions were 550 for *pmr1* and 800 $\mu E/m^2/s$ for *mtf1*.

(D) Confocal images of WT (without any fluorescent tag), and un-tagged Venus.

(E) Additional confocal images for Figures 2 and 6.

(F) Control for PMR1's indirect immunofluorescence shown in Figure 6Q. As expected, much-lower anti-PMR1 signal is observed in the *pmr1* mutant (LMJ.RY0402.206992).

CellPress
OPEN ACCESS

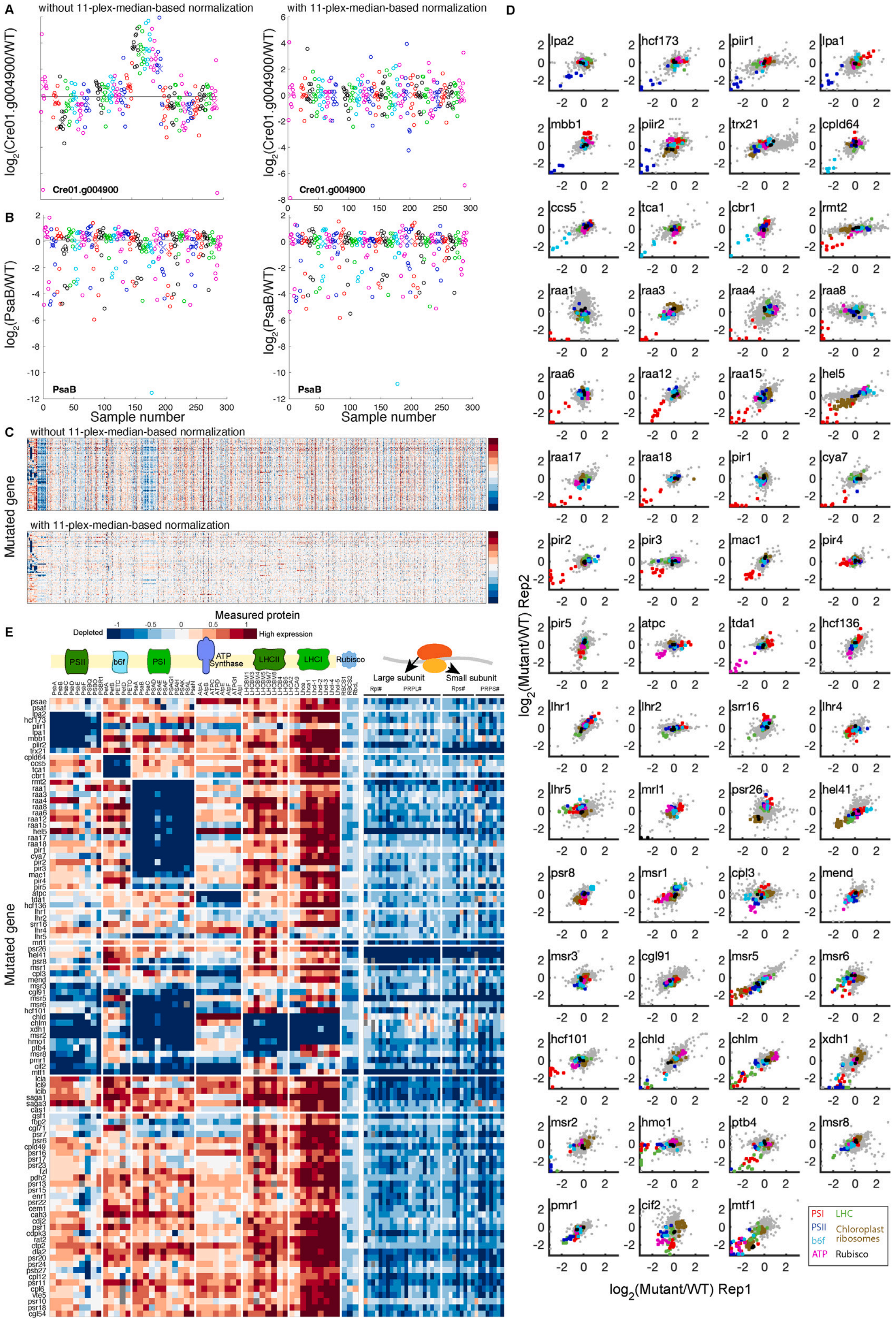

(legend on next page)

Figure S4. Normalization of the proteomic data, related to Figures 3 and 4

(A and B) Example of the data of two proteins (Cre01.g004900, A and PsaB, B) across all samples Without and with 11-plex-median-based normalization. Each proteomic 11-plex (10 samples and WT) is shown in one color, and the 11-plex's median is shown as an x in the same color. In addition to the samples included in Figure 4, these data include additional samples including controls and repeats that were subsequently filtered out due to noisy or contaminated samples. Without normalization, we can see systematic differences in protein abundance between the 11-plexes, which are removed by normalizing the protein's measured abundance using the group median, which we refer to as the 11-plex-median-based normalization. The black lines represent the median of all the samples.

(C) The normalization reduces the noise and systematic errors in the data. Protein levels are shown for proteins measured in at least 65% of the experiments characterizing the 100 mutants. The data are the average of two repeats on the $\log_2$ scale. The upper panel is before, and the lower panel is after the 11-plex-median-based normalization. We can see that the normalization removes much of the noise and maintains most of the signal. The left-most ~90 proteins are the ones shown in Figure 4.

(D) Scatterplots comparing the two replicate measurements of protein levels in the mutants shown in Figures 4B–4I.

(E) The data from Figure 4 are shown without the 11-plex-median-based normalization. A systematic underrepresentation of ribosomal proteins is apparent; we think this is due to a relative overrepresentation of ribosomal proteins in the reference wild-type strain that was included in the 11-plexes and that was used to initially normalize all raw data. As can be seen in Figure 4, the 11-plex-median-based normalization eliminates such systematic under- and overrepresentation.

CellPress
OPEN ACCESS

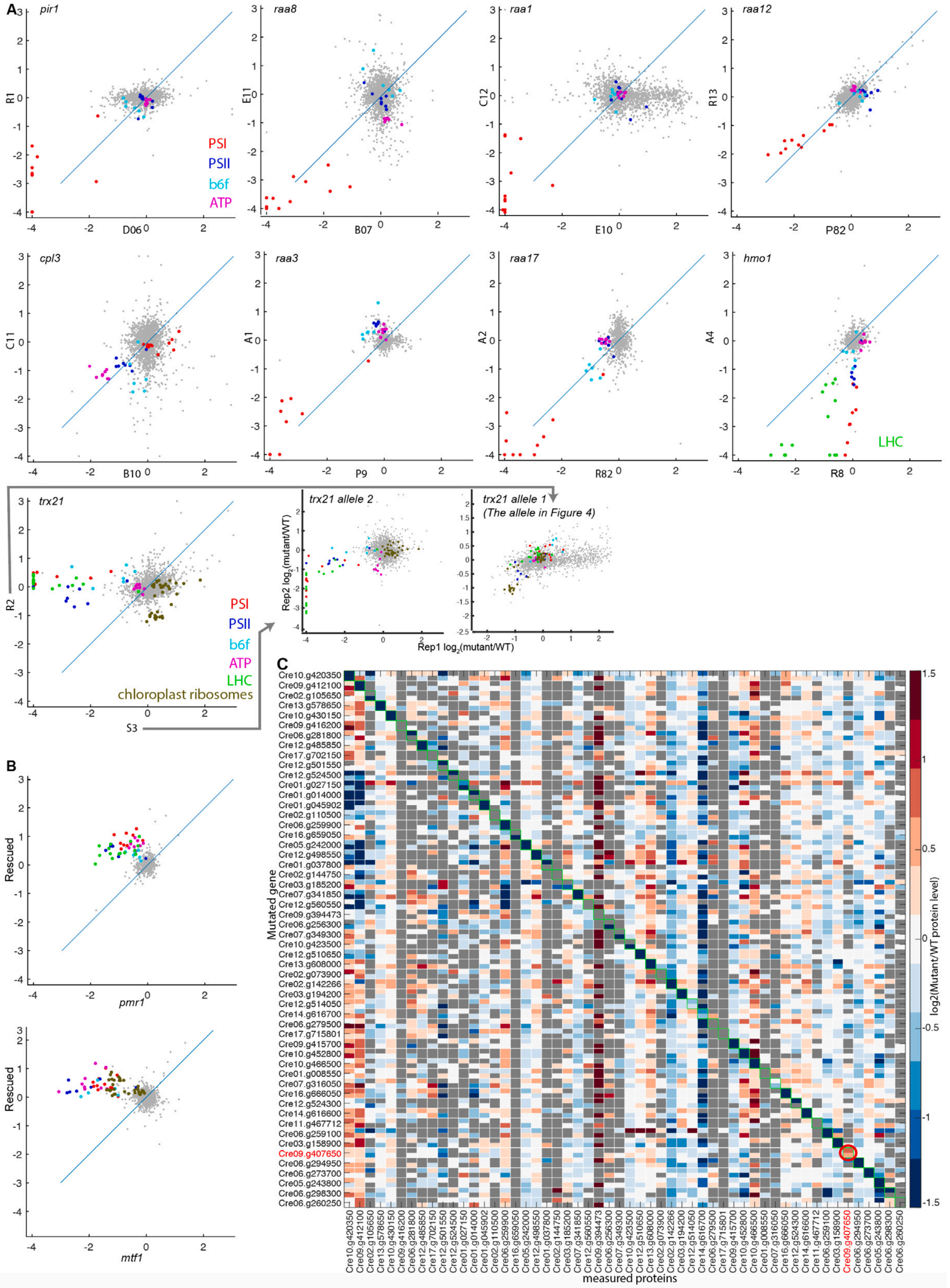

Figure S5. Proteomic controls, related to Figure 4

(A) Proteomes are compared between two different mutant alleles of the same gene. We only show data where we obtained data for both alleles and at least one of the alleles showed proteomic changes above the noise level. Each axis represents one allele's log_2(mutant/WT) proteomic data. The sample name is shown near each axis.

(B) Genes for which we rescued the mutants and collected proteomics data for both the mutants and the rescued strains. (A and B) Our data suggest that the impact on the photosynthetic complexes is from our mutant gene in all cases except for *TRX21*. The two *trx21* mutants have different phenotypes: one was yellow and had a decreased abundance of chlorophyll-binding proteins (including PSII), and the other was green and only affected PSII and the small subunit of the chloroplast ribosome. These observations suggest that the yellow mutant has an additional mutation leading to the additional proteomic phenotype. Additionally, 5 genes (HCF173, CPLD64, CHLM, RAA6, and RAA17) showed strong proteomic and photosynthetic phenotypes, and their rescue restored the mutant to WT-like growth. This demonstrates that only in rare cases (1/16) does the prominent proteomic phenotype come from a second mutation.

(C) Proteomic validation that the mutated protein is absent from the strains. We show the proteins' relative abundance without the 11-plex-median-based normalization (see Figure S4E). We observed downregulation of the mutated protein in all cases where we could measure the protein except for Cre09.g407650 (encircled in red), suggesting that Cre09.g407650 is a false positive. The insertion in Cre09.g407650 is in the 3′ UTR and was linked to the phenotype; this insertion is likely not the reason for the photosynthetic phenotype, demonstrating how proteomics can help identify false positives.

CellPress
OPEN ACCESS

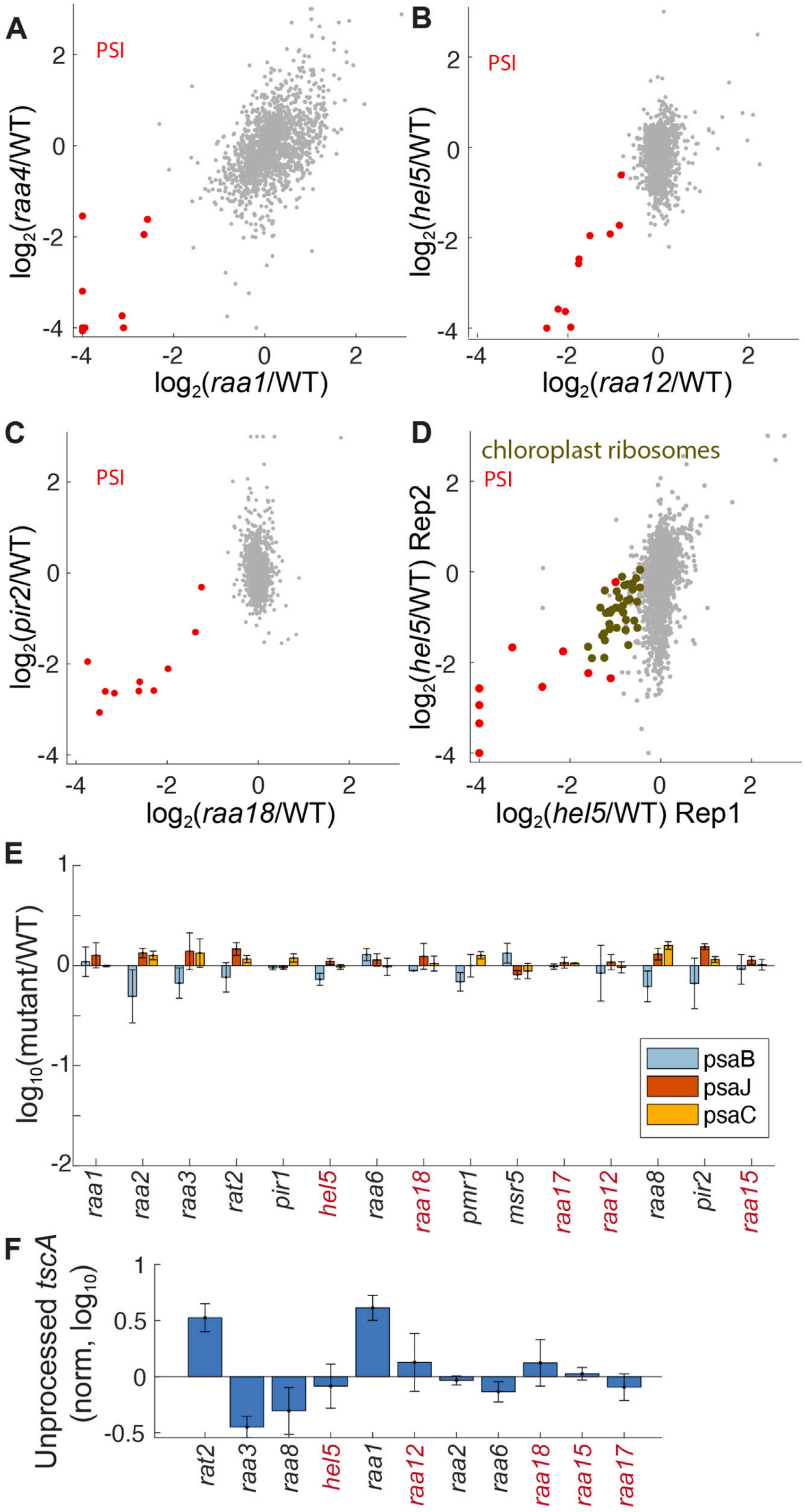

(legend on next page)

Figure S6. Supplemental data for *psaA* mRNA maturation factors, related to Figure 5

(A–C) Scatterplots of proteomic data in mutants in known *psaA* maturation factors (RAA1 and RAA4) and mutants in genes with similar proteomic profiles (HEL5, RAA12, PIR2, and RAA18). The data reflect the average normalized $\log_2$ (mutant/WT protein abundance) from two independent experiments.

(D) Scatterplot of two replicates of proteomic data of *hel5* mutants.

(E) The mRNA levels (normalized to WT) of *psaB*, *psaJ*, and *psaC* in the different mutants. The largest effects are 2-fold changes in *psaB* levels, which is within the noise level and is not expected to affect translation levels.[5] Error bars represent SE. The chloroplast mRNA does not have poly(A), so we used an rRNA depletion kit to remove most of the rRNA before amplifying RNA (see the "chloroplast transcriptome profiling (chloroplast RNA-seq)" section of the STAR Methods).

(F) RAT2 and RAA1 are required for *tscA* processing. This requirement suggests that *tscA* processing is carried out in conjunction with the splicing complex organized around RAA1.[48,58] Error bars represent SE.

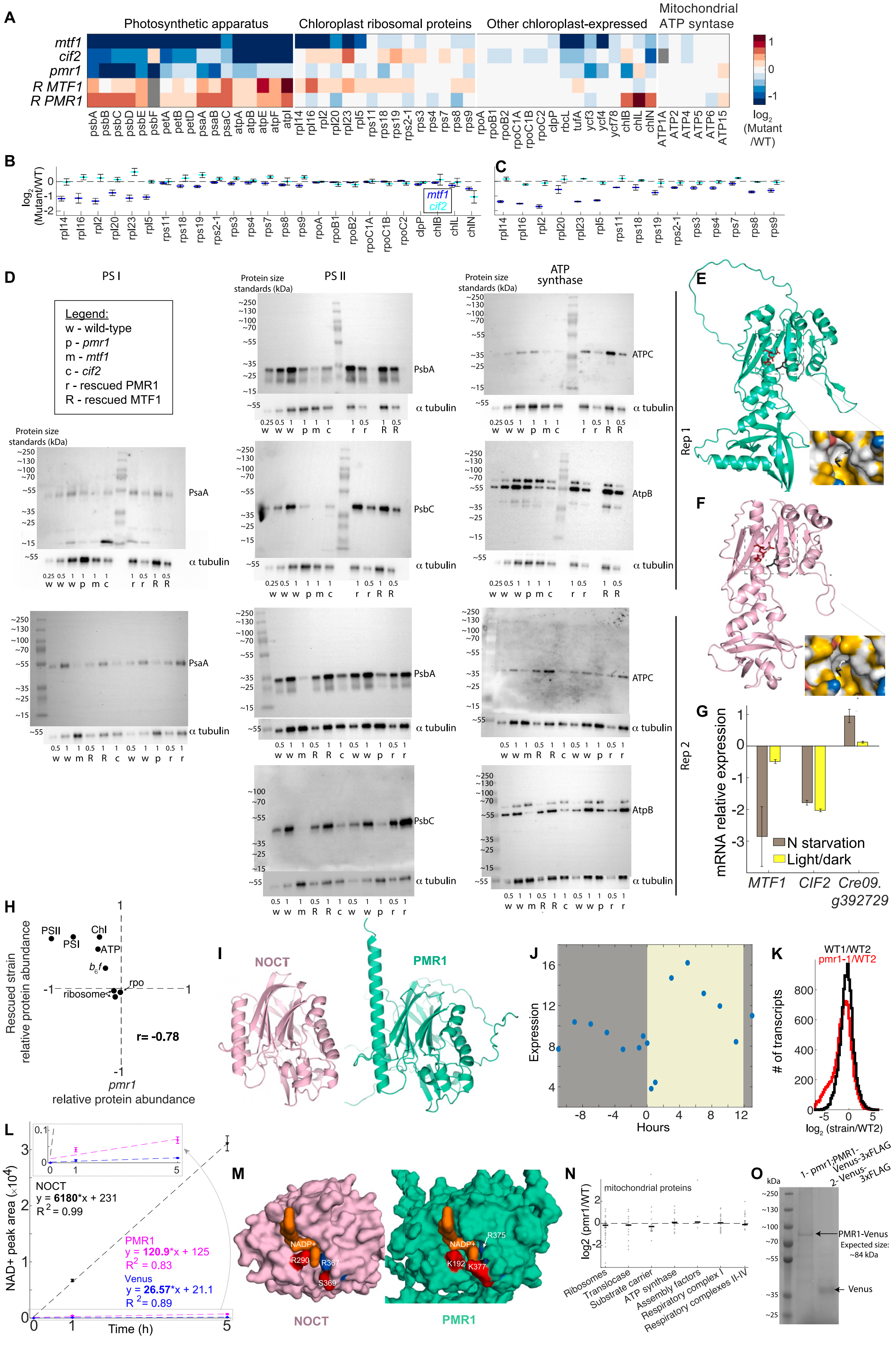

(legend on next page)

Figure S7. Supplemental data for MTF1, CIF2, and PMR1, related to Figure 6

(A) Protein levels of chloroplast-expressed genes and mitochondrial controls in *mtf1*, *cif2*, *pmr1*, rescued MTF1, and rescued PMR1. The data represent the median of 6, 3, 4, 4, and 3 independent experiments, respectively. Those replicates include the 2 experimental replicates shown in Figure 4 for *mtf1*, *cif2*, and *pmr1*, as well as additional experimental replicates.

(B) Detailed comparison of the chloroplast-expressed rpo RNA polymerase, rps and rpl ribosomal proteins, the clpP protease, and chl chlorophyll biosynthesis proteins between *mtf1* and *cif2* mutants. The median of 6 and 3 repeats ±SE is shown.

(C) Comparison of the chloroplast-expressed ribosomal proteins between *mtf1* and *cif2* mutants. The data in this control experiment are based only on two experimental repeats of each mutant that were run together in the same 11-plex to allow more-direct compression between them than the data shown in (B), which were obtained from different 11-plexes. Note that the data are very similar in both analyses.

(D) Images of the western blot membranes. We used each membrane to measure the relative abundance of one protein (using immunoblotting), then we used α-tubulin immunoblotting on the same membrane as a loading control (the image of the α-tubulin blot is shown below each membrane). We performed two experimental replicates with each antibody and used the second replicate for Figure 6. Note that the AtpB antibody recognizes not only the chloroplast's AtpB (~55 kDa) but also the mitochondrial ATP synthase beta subunit (~70 kDa); see the product information on Agrisera website for antibody AS05 085. For PsbA, a band with a lower molecular weight was observed when its antibody was used on *Chlamydomonas*; this is thought to be due to D1 degradation (see Agrisera website for antibody AS05 084A).

(E and F) Comparison between the AlphaFold-predicted MTF1 structure I and the crystal structure of *E. coli* MTF[64] (F). The conserved active-site residues (Asn108, His110, and Asp146 in *E. coli* MTF[64]; corresponding to Asn160, His162, and Asp198 in MTF1) are shown in red, and fMet is shown in black. For a better comparison of the active sites, we used YRB,[97] a script that displays the hydrophobic pockets (yellow) and negative charges (red) on a protein surface. In both active sites, we can see hydrophobic pockets below the fMet and negatively charged active-site residues above it.

(G) Expression data[41,98] for *MTF1*, *CIF2*, and *Cre09.g392729* (encoding the mitochondrial predicted MTF1 ortholog) are shown for different growth conditions.

(H) There is a strong negative correlation (r = −0.78) between the levels of chloroplast-expressed complexes in the *pmr1* mutant and their levels in the PMR1 rescued strain, supporting the idea that PMR1 overexpression in the rescued strain leads to the overexpression of chloroplast-expressed complexes.

(I) Comparison between the AlphaFold-predicted PMR1 structure (green) and the crystal structure of human nocturnin (pink).[99]

(J) PMR1 diurnal expression. The light period is shown in yellow, and the dark period is shown in gray. The data are from Strenkert et al.[71]

(K) The *pmr1* mutant affects the mRNA expression of many genes. The $\log_2$(pmr*1-1*/WT2) data are shown in red, and as control, the $\log_2$(WT1/WT2) data are shown in black.

(L) PMR1 $NADP^+$ phosphatase activity *in vitro*. We started with $NADP^+$ and used LC-MS to follow the accumulation over time of NAD^+ after the addition of one of the following proteins: NOCT (positive control), PMR1-Venus-3xFLAG, or Venus-3xFLAG (negative control—contaminants from the IP may contribute phosphatase activity). The data represent the median of three replicates ±SE. For each protein we calculated a linear fit using MATLAB's "polyfit" command. The linear fits' slopes (normalized by 61.8), and their SE (calculated by MATLAB's "fitlm" command) were used to generate Figure 6I.

(M) Comparison of the substrate-binding pockets between the NOCT structure (pink) and AlphaFold-predicted PMR1 structure (green). $NADP^+$ is illustrated in orange. Changing R290 to K192 affects the shape of the substrate-binding region, and lysine K377 may partially block the entrance to the substrate-binding pocket in PMR1.

(N) The *pmr1* mutant does not lead to the downregulation of mitochondrial gene mRNAs. Each dot represents the mean of 2 experimental replicate measurements of one gene. The bar represents the median value across all genes in a group.

(O) The predominant form of PMR1 in the rescued *pmr1*;PMR1-Venus-3xFLAG strain is of the expected molecular weight. We performed an anti-FLAG immunoprecipitation from (1) the rescued *pmr1*;PMR1-Venus-3xFLAG strain and (2) a strain expressing Venus-3xFLAG. The products were run on a gel and stained with Coomassie (EZBlue gel).
